# Estimation of reference curves for brain atrophy and analysis of robustness to machine effects

**DOI:** 10.1038/s41598-025-18073-z

**Published:** 2025-10-03

**Authors:** Elodie Piot, Félix Renard, Arnaud Attyé, Alexandre Krainik

**Affiliations:** 1 Functional neuroimaging and cerebral perfusion laboratory, Grenoble Institute of Neurosciences (GIN, Grenoble Alpes University), Grenoble, France; 2 GeodAIsics, Grenoble, France; 3 University Grenoble Alps, 38000 Grenoble, France; 4 University Grenoble Alpes, Inserm, CHU Grenoble Alpes, CNRS, IRMaGe, 38000 Grenoble, France; 5 Department of neuroradiology, University Hospital of Grenoble, 38000 Grenoble, France; 6 UC San Francisco, San Francisco, CA 94107 USA; 7 UC San Diego, La Jolla, CA 92093 USA; 8 Mayo Clinic, Rochester, Mn USA; 9 UC Berkeley, Berkeley, San Francisco, USA; 10 University of Pennsylvania, Philadelphia, PA 19104 USA; 11 USC, Los Angeles, CA 90032 USA; 12 UC Davis, Sacramento, CA USA; 13 Brigham and Women’s Hospital/Harvard Medical School, Boston, MA 02215 USA; 14 Indiana University, Bloomington, IN 47405 USA; 15 Washington University St. Louis, St. Louis, MO 63110 USA; 16 Prevent Alzheimer’s Disease 2020, Rockville, MD 20850 USA; 17 Siemens, Erlangen, Germany; 18 Alzheimer’s Association, Chicago, IL 60631 USA; 19 University of Pittsburgh, Pittsburgh, PA 15213 USA; 20 Cornell University, Ithaca, NY 14853 USA; 21 Albert Einstein College of Medicine of Yeshiva University, Bronx, NY 10461 USA; 22 AD Drug Discovery Foundation, New York, NY 10019 USA; 23 Acumen Pharmaceuticals, Livermore, CA 94551 USA; 24 Northwestern University, Chicago, IL 60611 USA; 25 National Institute of Mental Health, Bethesda, MD 20892 USA; 26 Brown University, Providence, RI 02912 USA; 27 University of Washington, Seattle, WA 98195 USA; 28 University of London, London, UK; 29 UC LA, Torrance, CA 90509 USA; 30 University of Michigan, Ann Arbor, MI 48109-2800 USA; 31 University of Utah, Salt Lake City, UT 84112 USA; 32 Banner Alzheimer’s Institute, Phoenix, AZ 85006 USA; 33 UUC Irvine, Orange, CA 92868 USA; 34 Johns Hopkins University, Baltimore, MD 21205 USA; 35 Richard Frank Consulting, Consulting, New York, USA; 36 National Institute on Aging, Baltimore, Maryland USA; 37 Oregon Health and Science University, Portland, OR 97239 USA; 38 University of Alabama, Birmingham, AL USA; 39 Mount Sinai School of Medicine, New York, NY USA; 40 Rush University Medical Center, Chicago, IL 60612 USA; 41 Baylor College of Medicine, Houston, TX USA; 42 Wien Center, Miami Beach, FL 33140 USA; 43 Columbia University Medical Center, New York, NY USA; 44 New York University, New York, NY USA; 45 University of Texas Southwestern Medical School, Galveston, TX 77555 USA; 46 Duke University Medical Center, Durham, NC USA; 47 Emory University, Atlanta, GA 30307 USA; 48 University of Kansas Medical Center, Kansas City, Kansas USA; 49 University of Kentucky, Lexington, KY USA; 50 Mayo Clinic, Jacksonville, Florida USA; 51 University of Rochester Medical Center, Rochester, NY 14642 USA; 52 Yale University School of Medicine, New Haven, ct USA; 53 McGill Univ. Montreal-Jewish General Hospital, Montreal, PQ H3A 2A7 Canada; 54 Sunnybrook Health Sciences, Toronto, On Canada; 55 U.B.C. Clinic for AD and Related Disorders, Vancouver, BC Canada; 56 Cognitive Neurology - St. Joseph’s, London, ON Canada; 57 Cleveland Clinic Lou Ruvo Center for Brain Health, Las Vegas, NV 89106 USA; 58 Premiere Research Inst (Palm Beach Neurology), W Palm Beach, FL USA; 59 Georgetown University Medical Center, Washington, DC, 20007 USA; 60 Stanford University, Stanford, CA 94305 USA; 61 Boston University, Boston, Massachusetts USA; 62 Howard University, Washington, DC 20059 USA; 63 Case Western Reserve University, Cleveland, OH 44106 USA; 64 Neurological Care of CNY, Liverpool, NY 13088 USA; 65 St. Joseph’s Health Care, London, ON N6A 4H1 Canada; 66 Dent Neurologic Institute, Amherst, NY 14226 USA; 67 Ohio State University, Columbus, OH 43210 USA; 68 Albany Medical College, Albany, NY 12208 USA; 69 Hartford Hospital Olin Neuropsychiatry Research Center, Hartford, CT 06114 USA; 70 Dartmouth-Hitchcock Medical Center, Lebanon, NH USA; 71 Wake Forest University Health Sciences, Winston-Salem, NC USA; 72 Medical University South Carolina, Charleston, SC 29425 USA; 73 Nathan Kline Institute, Orangeburg, NY USA; 74 University of Iowa College of Medicine, Iowa City, IA 52242 USA; 75 University of South Florida, USF Health Byrd Alzheimer’s Institute, Tampa, FL 33613 USA; 76 University of California, San Francisco, USA; 77 Harvard Medical School and Massachusetts General Hospital, Boston, USA; 78 University of Washington School of Medicine, Seattle, USA; 79 Mayo Clinic, Rochester, USA

**Keywords:** Reference curves, AssemblyNet, FastSurfer, FreeSurfer, Robustness, Atrophy, Sensitivity, Learning algorithms, Nervous system, Neurodegeneration, Diagnostic markers, Dementia, Neurodegeneration, Neurodegenerative diseases, Biomedical engineering, Scientific data, Software

## Abstract

Neurodegenerative diseases like Alzheimer’s are difficult to diagnose due to brain complexity and imaging variability. However, volumetric analysis tools, using reference curves, help detect abnormal brain atrophy and support diagnosis and monitoring. This study evaluates the robustness of three segmentation algorithms, AssemblyNet, FastSurfer and FreeSurfer, in constructing brain volume reference curves and detecting hippocampal atrophy. Using data from 3,730 cognitively normal subjects, we built reference curves and assessed robustness to magnetic field strength (1.5T vs. 3T) using four error metrics (sMAPE, sMSPE, wMAPE, sMdAPE) with bootstrap validation. We evaluated classification performance using hippocampal atrophy rates and HAVAs scores (Hippocampal-Amygdalo-Ventricular Atrophy scores). AssemblyNet shows the lowest errors across all robustness metrics. In contrast, FastSurfer and FreeSurfer exhibit greater deviations, indicating higher sensitivity to field strength variability. AssemblyNet provides consistent hippocampal atrophy rates across all reference models, despite slightly lower sensitivity, while FastSurfer and FreeSurfer display greater variability. Specificity ranges from 0.87 to 0.91 for AssemblyNet, compared to 0.76-0.93 for FastSurfer and 0.86-0.93 for FreeSurfer. Using the HAVAs score, all methods detect high atrophy rates in Alzheimer’s patients. FastSurfer achieves the highest sensitivity (0.98), while AssemblyNet reaches the best specificity (0.95) and the highest balanced accuracy (0.91). This study underscores the importance of algorithm choice for reliable brain volumetric analysis in heterogeneous imaging environments. Among the methods tested, AssemblyNet stands out as both sensitive to Alzheimer’s-related atrophy and robust to acquisition variability, making it a strong candidate when analyzing hippocampal volumes in large, multi-site datasets.

## Introduction

Neurodegenerative diseases cause accelerated neuronal death, leading to varying levels of cerebral atrophy^1^. The spatial pattern of atrophy provides critical insights for differential diagnosis and disease staging^2^^,^^3^. To better quantify and interpret such patterns, normative modeling tools have emerged as valuable resources. These tools rely on reference curves that represent healthy age-related variation in brain structure, allowing for the identification of individual deviations from typical trajectories^4^^,^^5^^,^^6^. By comparing an individual’s brain volumes to these normative models, deviations indicative of abnormal atrophy can be detected. In the context of neurodegenerative disease, atrophy is typically defined when regional volumes fall below the lower bounds of these normative distributions, providing a standardized and interpretable framework for assessing structural brain changes^7^.

However, the increasing use of large, diverse datasets has introduced additional challenges. Modern research relies on databases of unprecedented size, incorporating data from a wide variety of sources (Coupe et al., (2023)^4^: 40,944 subjects pooled across 24 databases). This diversity increases the prevalence of the “machine effects”, where differences in MRI acquisition parameters^8^, scanner hardware^9^, and processing pipelines^10^ may lead to biases in volumetric measurements. Such variability can mask subtle disease-related changes, as differences in MRI acquisition parameters alone can alter volume measures by up to 4.8%^11^, comparable to early disease-related brain volume changes^12^.

Tools for volume-based diagnostics enable clinicians to monitor disease progression and evaluate severity, with atrophy serving as a reliable biomarker^14^^,^^15^. However, the impact of the machine effects underscores the need for robust algorithms. Commercial tools for volume-based diagnostics have emerged to meet increasing demand^16^^,^^17^^,^^18^, yet most lack thorough validation: of 17 identified products, only 4 underwent clinical validation in dementia populations^19^. Moreover, normative datasets vary widely (100-8,000 subjects), raising concerns about reliability and generalizability^19^. Further validation is essential for their full potential in neurodegenerative disease diagnosis and monitoring.

Several studies propose reference curves with divergent trajectories for cortical and subcortical structures, sometimes described as linear, U-shaped, or complex polynomial curves, highlighting a lack of consensus in the field^20^. This variability has led to inconsistent findings^21^ and, in some cases, may even alter the observed differences between control and pathological groups^22^. As explained by Coupe et al., (2017)^20^, several factors contribute to these inconsistencies: the use of data covering only restricted age ranges, which biases the construction of reference curves, limited scanner diversity within certain age groups^19^^,^^23^, non-harmonized acquisition protocols^24^, differences in curve modeling approaches and segmentation tools^20^^,^^23^ and the use of heterogeneous volumetric measures (e.g., absolute volumes, normalized volumes, or z-scores)^25^.

Selecting a model for normative curves is challenging, requiring a balance between flexibility and overfitting (where the model becomes too tailored to the training data and fails to generalize to new data)^23^^,^^26^. Options range from linear models^20^ to advanced methods like GAMLSS^27^. This study uses Generalized Additive Models (GAMs) to test segmentation reproducibility with a standard approach. GAMs extend generalized linear models, offering flexibility and controlling overfitting through constraints^28^^,^^29^.

Normative curve robustness is as crucial as model selection. Liu et al., (2024)^24^ show that identical models yield different curves across datasets. Sample size, age representation, and intracranial volume normalization further impact results^20^. Temporal dependencies and machine effects also challenge reproducibility, underscoring the need for robust methods.

Several methods exist for comparing reference curves. Summing pairwise distances is simple but prone to error cancellation. Advanced time series metrics (please refer to Supplementary Section S1) provide a more robust assessment by capturing trajectory alignment. These metrics, available in classical, symmetrized, and sometimes weighted forms, were chosen for their interpretability, consistency, scale invariance, and balanced handling of over- and under-predictions.

**Fig. 1:**
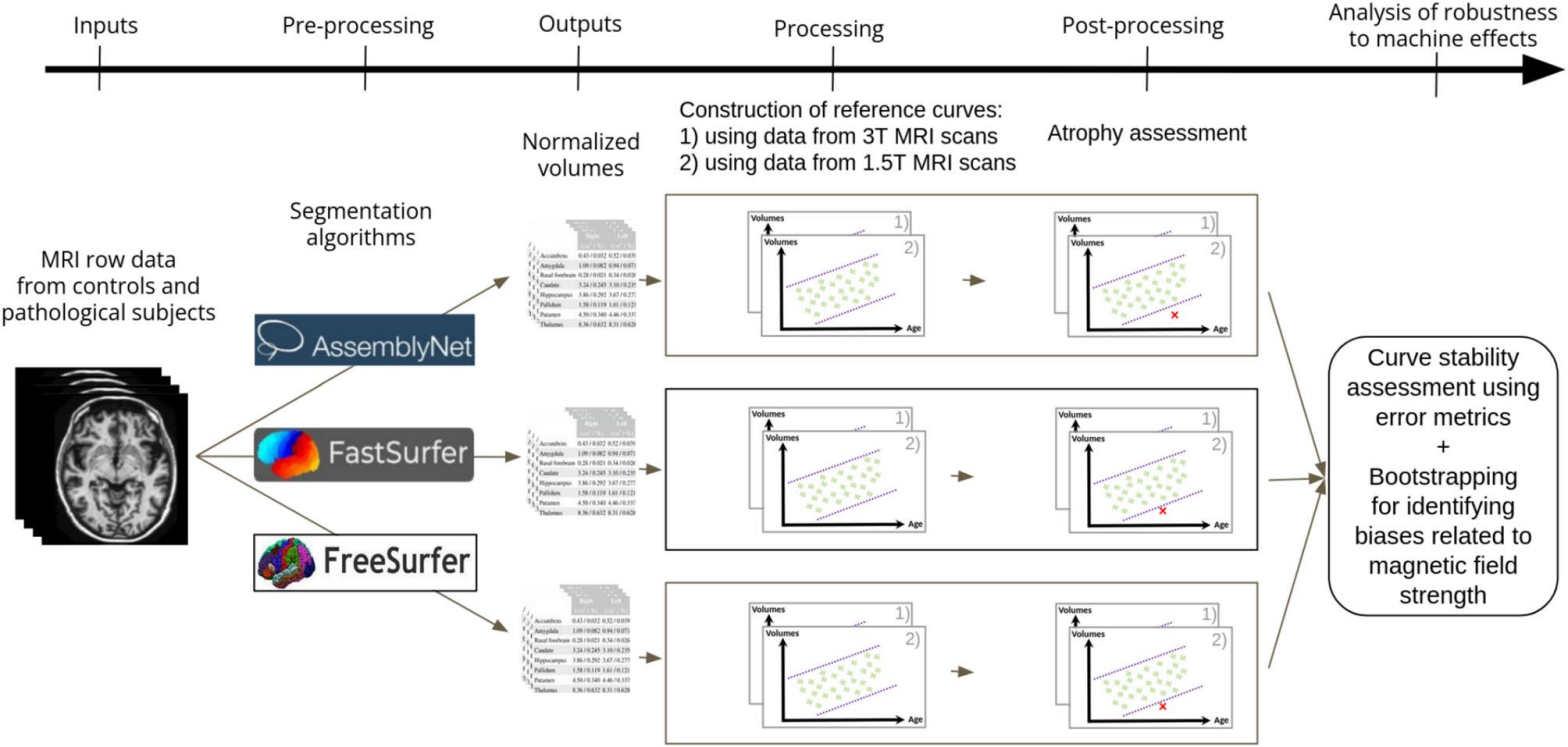
Study overview.

The first objective of this study is to investigate the robustness of AssemblyNet, FastSurfer and FreeSurfer in generating reference curves and evaluating brain atrophy using GAMs. The second objective is to develop a methodology for comparing reference curves estimated from volumetric data of 3,730 healthy subjects. These curves, generated by three segmentation algorithms, that extract brain volumes, are evaluated for robustness across magnetic field strengths. To our knowledge, no study has used GAMs to build reference curves for AssemblyNet, FastSurfer, and FreeSurfer, quantified segmentation algorithm impact, or assessed robustness to machine effects. The third objective is to assess segmentation algorithms’ sensitivity and stability by measuring the proportion of Alzheimer’s patients (AD) with hippocampal atrophy^30^^,^^31^ and HAVAs scores^32^ (Hippocampal-Amygdalo-Ventricular Atrophy scores); and comparing results with the literature. The Scheltens scale^33^ is commonly used but observer-dependent^34^. While visual assessment detects cerebrospinal fluid enlargement, it only indirectly reflects gray or white matter loss. Automated segmentation directly targets gray matter, overcoming this limitation. To our knowledge, the impact of machine effects on atrophy assessment using GAMs remains unexplored. For an overview of this study, please refer to Fig. 1.

## Materials and methods

### Data

#### Data used to construct reference curves

To build our reference curves, we segmented 3730 T1-weighted MRI scans of healthy subjects from 11 open-access datasets: ABIDE^35^ (n=469), ICBM^36^ (n=294), IXI (https://brain-development.org/ixi-dataset/) (n=549), ADNI^37^ (n=373), OASIS1^38^ (n=298), PPMI^39^ (n=166), UCLA^40^ (n=125), DLBS (https://sites.utdallas.edu/dlbs/) (n=315), SALD^41^ (n=494), NIFD (http://memory.ucsf.edu/research/studies/nifd) (n=114), and SLIM (https://fcon_1000.projects.nitrc.org/indi/retro/southwestuni_qiu_index.html) (n=580).

**Fig. 2:**
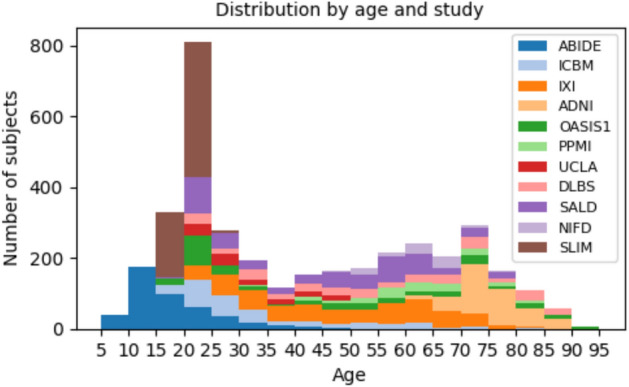
Datasets used to construct our reference curves: distribution by age and study.

Figure 2 illustrates the distribution by age and study. Table 1 show a summary of the key characteristics of the 11 datasets presented.

Part of the data for this work were sourced from the International Consortium for Brain Mapping (ICBM) dataset (https://www.loni.usc.edu/). Then, part of the data utilized in this study were sourced from the Alzheimer’s Disease Neuroimaging Initiative (ADNI) dataset (adni.loni.usc.edu). Established in 2003 as a public-private partnership and led by Principal Investigator Michael W. Weiner, MD, ADNI’s primary objective is to evaluate whether the combination of serial MRI, positron emission tomography (PET), various biological markers, and clinical and neuropsychological assessments can track the progression of mild cognitive impairment (MCI) and early Alzheimer’s disease (AD). For the most recent updates, please visit www.adni-info.org. In addition, we used data from the Open Access Series of Imaging Studies (OASIS) OASIS1, which provides cross-sectional MRI data in young, middle-aged, nondemented, and demented older adults. Finally, NIFD is the abbreviation for the frontotemporal lobar degeneration neuroimaging initiative (FTLDNI).

**Table 1:** Main characteristics of the datasets used to construct the reference curves. N_sub: number of subjects; min/max/mean/std: age statistics; F/M: number of females/males; 1.5T/3T: number of scans acquired at each field strength.

**Dataset**	**N**_**sub**	**Age**				**Sex**		**Acquisition and Field Strength**		
		Min	Max	Mean	Std	F	M	Manufacturer	1.5T	3T
ABIDE	448	7	56	17	7.7	18.5%	81.5%	GE/Philips/Siemens	0	448
ICBM	294	18	80	34	14.3	48.3%	51.7%	Philips/Siemens	278	16
IXI	549	19	86	48	16.5	55.9%	44.1%	Philips/GE	371	178
ADNI	372	56	90	75	5.9	51.6%	48.4%	GE/Philips/Siemens	193	179
OASIS	298	18	94	45	23.8	62.8%	37.2%	Siemens	298	0
PPMI	150	31	83	60	11.5	34.7%	65.3%	GE/Philips/Siemens	38	112
UCLA	125	21	50	32	8.8	47.2%	52.8%	Siemens	0	125
DLBS	314	20	89	54	20.0	62.7%	37.3%	Philips	0	314
SALD	494	19	80	45	17.4	62.1%	37.4%	Siemens	0	494
NIFD	112	47	81	63	7.2	55.4%	44.6%	Siemens	0	112
SLIM	574	17	27	20	1.3	55.7%	44.3%	Siemens	0	574
**Total**	**3730**	**7**	**94**	44	12.2	51.2%	48.8%	GE/Philips/Siemens	**1178**	**2552**

**Table 2:** Main characteristics of the datasets used for sensitivity and stability analysis. N_sub: number of subjects; min/max/mean/std: age statistics; F/M: number of females/males; 1.5T/3T: number of scans acquired at each field strength; CN: controls subjects; AD: Alzheimer’s patients.

**Dataset**	**N**_**sub**	**Age**				**Sex**		**Field Strength**		**Analysis**
		Min	Max	Mean	Std	F	M	1.5T	3T	
ADNI AD	219	65	80	74.1	4.0	48.9%	51.1%	102	117	Sensitivity
ADNI CN	255	65	80	71.7	4.2	58.8%	41.2%	24	231	Sensitivity
MIRIAD AD	46	55	85	68.7	7.1	58.7%	41.3%	46	0	Stability
MIRIAD CN	22	58	85	68.9	7.2	50%	50%	22	0	Stability
SRPBS	9	24	32	27	2.6	0%	100%	0	9	Stability

#### Data for sensitivity and stability analysis

To evaluate the sensitivity of segmentation algorithms, we segmented images of 219 AD subjects and 255 controls subjects from ADNI (https://adni.loni.usc.edu/). The subjects are all between 65 and 80 years of age.

For the stability analysis, we used the data of 46 patients with “mild-moderate Alzheimer’s disease” and 22 age-matched healthy subjects from Malone et al., (2013)^42^ (MIRIAD). They have been scanned 8 times (2, 6, 14, 26, 38 and 52 weeks, 18 and 24 months from baseline) on the same 1.5 T scanner. Additionally, stability was assessed using the data of 9 healthy traveling subjects from Tanaka et al., (2021)^43^ (SRPBS). They have been scanned at 12 different imaging centers within a 30-day period, making up a total of 156 exams. Three MRI manufacturers, and seven MRI scanner types were used (3T only). Table 2 presents the dataset used for the sensitivity and stability analysis of segmentation algorithms.

### Pipeline analysis

#### Segmentation algorithms

The data were segmented using AssemblyNet (version 1.0.0), a segmentation algorithm based on a large ensemble of Convolutional Neural Networks (CNNs)^44^. Segmentation was then performed using 250 deep learning models through a multiscale framework.

We compared our results with FreeSurfer, one of the most widely used segmentation software packages in neuroimaging^45^. All data was processed using FreeSurfer version 7.3.1. We segmented each subject using the automated “recon-all” pipeline with the default parameters. For FreeSurfer, because of the time required for segmentation (around 15 h per subject), the FreeSurfer segmentations were calculated on the VIP platform, which provides substantial computing resources^46^.

FastSurfer^47^(version 2.4.2) builds upon FreeSurfer and incorporates technologies similar to those used in AssemblyNet (Deep Learning), enabling segmentation in approximately ten minutes. FastSurfer utilizes three Fully Convolutional Neural Networks (F-CNNs), each responsible for segmenting 2D slices in the coronal, axial, and sagittal planes. The three segmentations are then aggregated.

#### GAM and constraints

Reference curves were estimated using a Generalized Additive Models (GAM) model^48^(pyGAM (ExpectileGAM) version 0.9.1). This model generates a curve fitting a set of points using a flexible combination of smooth functions, and captures linear relationships between points. GAM’s model extends generalized linear models resulting in a highly flexible model, in which it is easy to control overfitting.

To prevent overfitting and avoid “non-physical” behaviors, such as abrupt variations in the variable over short periods throughout life, we applied convex or concave smoothing techniques. Overfitting can result from excessive learning from data, leading to a strong influence from specific studies rather than generalizable patterns. By applying appropriate smoothing methods, we ensure a more physiologically plausible representation of changes over time. Supplementary Section S2, Fig. S1 illustrates smoothing effects.

### Curve stability assessment

#### Methods for comparing reference curves across magnetic field strengths

Results in the “Assessment of curves stability” section include visual and quantitative analyses: the former offers a graphical comparison, while the latter quantifies visual differences using different methods. We assessed the robustness of segmentation algorithms to magnetic field strength variations by comparing reference curves derived from 1.5T data with those from 3T data. For this analysis, we compared the lower, mean, and upper bound curves using metrics designed to quantify differences between curve pairs.

**Measure of errors between curves**

We aimed to compare reference curves that depict volume evolution over a lifespan. To achieve this, we explored metrics for time series analysis and forecasting that calculate errors between these curves. Given the nature of our data, the selected metrics had to meet specific requirements: 1) Interpretability: the metrics should be easy to interpret, for example, by being on the same scale as the data or presented as a percentage. 2) Independence from error sign: the metric should not cancel out positive and negative errors. This means the metric should not differentiate between the direction of the error. 3) Scale-independence/invariance: the metric should remain consistent regardless of data scaling, allowing comparisons between different algorithms. 4) Treat over-predictions and under-predictions equally.

Based on the literature, we selected 18 error metrics for evaluating the reference curves. An overview of these metrics is provided in Supplementary Section S1, where we define each metric, and discuss its strengths and limitations. We summarized the characteristics of the 18 metrics in relation to the selection criteria in Supplementary Section S1, Table S1. This table supports our selection of 5 key metrics for the comparison of reference curves: the symmetric Mean Squared Percentage Error (sMSPE^49^, sktime version 0.34.0), the Mean Absolute Scaled Error (MASE^49^, sklearn 1.4.1), the symmetric Mean Absolute Percentage Error (sMAPE^50^), the weighted Mean Absolute Percentage Error (wMAPE^51^) and the symmetric Median Absolute Percentage Error (sMdAPE^49^, sktime version 0.34.0). Since these are error metrics, the ideal value for all of them is 0, indicating no difference between reference curves.

Each metric has specific characteristics and relevance to our analysis, as described below: sMSPE amplifies larger errors by squaring the percentage differences, providing insight into the presence of significant deviations. sMAPE quantifies the percentage difference between predicted and reference values, taking into account both over- and under-predictions. A lower sMAPE indicates higher agreement between the predicted and reference values. wMAPE evaluates the absolute percentage errors while weighting them by the magnitude of the observed (reference) values. wMAPE accounts for errors weighted by volume size, making it crucial for datasets with a wide range of volumes. Since wMAPE accounts for volume size, this metric is particularly useful in applications where larger volumes might dominate the error. sMdAPE focuses on the median of absolute percentage differences, making it less sensitive to outliers.

**Method for identifying biases related to magnetic field strength: bootstrapping**

To evaluate the presence of bias related to the magnetic field strength, we compared the metric values (sMSPE, MASE, sMAPE, wMAPE and sMdAPE) obtained using the true labels with the distributions generated through bootstrapping^52^. We applied bootstrapping to evaluate the variability of error metrics under different configurations of shuffled labels. This approach helps to identify biases in the results.

Initially, we constructed two types of reference curves 1) using data from 1.5T MRI scans and 2) using data from 3T MRI scans. For each curve boundary, we calculated the metrics to compare the 1.5T curves against the 3T curves. Then, we performed a bootstrapping procedure^52^ involving 10,000 iterations. In each iteration, we shuffled the labels of the data, effectively randomizing the assignment of MRI scan data to the “1.5T” and “3T” groups. Using these shuffled labels, we reconstructed the reference curves for both groups and recalculated the metrics (sMSPE, MASE, sMAPE, wMAPE and sMdAPE) between the new curves. This allowed us to assess the variability in the metrics under random label assignments. The 5th percentile and 95th percentile were computed to characterize the range of variability.

To assess magnetic field bias, we compared the true-label metric values to the bootstrapped distributions. A significant bias was considered present if the true-label metric values fell outside the 5th-95th percentile range of the bootstrapped distributions. This would indicate that the observed differences between 1.5T and 3T data are not due to random variability but are likely influenced by differences related to magnetic field strength.

### Atrophy assessment

While reproducibility is crucial for a segmentation algorithm, it is ineffective if it cannot detect pathological variations. So, we examined how segmentation algorithms affect atrophy assessment by studying the sensitivity and stability of results across different magnetic field strengths.

#### Sensitivity analysis

To assess sensitivity, two complementary approaches were used. First, hippocampal atrophy percentages were computed for both AD patients and cognitively normal (CN) subjects. Second, we implemented the HAVAs method, from Coupe et al., (2022)^32^. In this method, hippocampal, amygdalar, and inferior lateral ventricle volumes were first normalized by intracranial volume^53^, then converted into z-scores using the mean and standard deviation from a reference set of 3730 CN subjects covering the full lifespan. This double normalization accounts for inter-individual and inter-structural variability^32^. The HAVAs score was then computed as the sum of the hippocampal and amygdalar z-scores, from which the z-scores of the inferior lateral ventricle is subtracted, based on the observation that AD is associated with atrophy in the hippocampus and amygdala and enlargement of the ventricle. Left and right HAVAs scores were also z-normalized using the same reference population.

#### Performance metrics

To evaluate the effectiveness of each segmentation method in distinguishing between AD and CN subjects, we computed standard classification metrics. These include sensitivity (or true positive rate), specificity (or true negative rate), balanced accuracy (the average of sensitivity and specificity), and the area under the ROC curve (AUC). A higher AUC indicates better discrimination between the two groups (AD vs. CN). Values closer to 1 reflect excellent performance, while values near 0.5 indicate performance close to chance. AUC values above 0.80 are typically considered clinically meaningful^54^. These metrics provide insight into the algorithm’s ability to correctly classify both group.

## Results

This study presents results with volumes normalized by the intracranial volume (ICV).

### Curve stability assessment

**Fig. 3:**
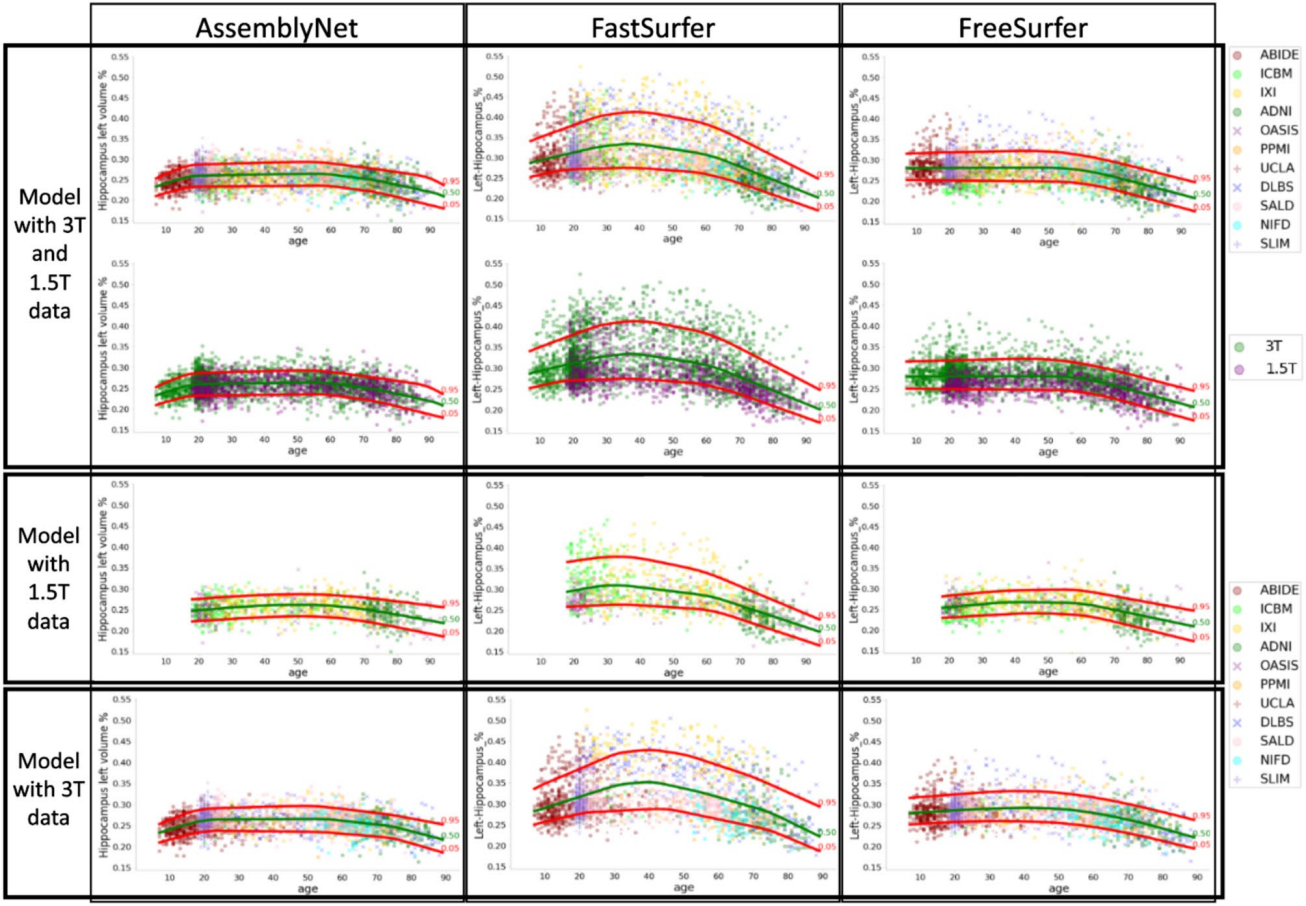
Reference curves for the left hippocampus (using concave smoothing), computed using data from different magnetic field strengths: 1.5T + 3T combined (first row: points colored by study; second row: points colored by field strength), only 1.5T (third row), and only 3T (fourth row). Each column corresponds to one of the three segmentation algorithms evaluated in the study. Each dot represents one subject. The 5th (red), 50th (green), and 95th (red) expectiles were computed to characterize the range of variability. AssemblyNet appears less sensitive to magnetic field strength variations compared to FastSurfer and FreeSurfer.

The first row of Fig. 3 presents reference curves for the left hippocampus, with curves for other regions available on https://gitlab.com/geodaisics1/ Estimation_of_reference_curves_for_brain_atrophy_and _analysis_of_ro bustness_to_machine_effects. The differences in FastSurfer’s reference curves compared to AssemblyNet and FreeSurfer suggest that using FastSurfer may yield varying results, potentially providing different information and influencing final diagnoses.

#### Evaluation of the consistency of reference curves across magnetic field strengths

**Preliminary results** We calculated the median percentage volume difference of 3730 subjects segmented by AssemblyNet, FastSurfer, and FreeSurfer, both with and without normalization by ICV (Supplementary Section S3, Fig. S2). For non-normalized volumes, the median percentage differences were 2.53% for AssemblyNet, 5.60% for FastSurfer, and 6.15% for FreeSurfer. The median percentage differences were 2.54% for AssemblyNet, 10.38% for FastSurfer, and 9.05% for FreeSurfer when normalized by the ICV.

**Visual analysis** Observing the points in the first row of Fig. 3, we noticed a greater dispersion of data for FastSurfer compared to the other two algorithms. To analyze the organization of these dots, especially for FastSurfer, and to determine if their positions are influenced by an acquisition parameter, we therefore repeated the first row of Fig. 3 but color-coding the points according to the magnetic field strength of the MRI machine in which the images were acquired. This is shown in the first line of Fig. 3. We then built additional reference curves using only the data from images acquired at 1.5T, shown in the second line of Fig. 3, and only the images acquired at 3T, shown in the third line of the same figure, for all three segmentation algorithms in the study. Visually, a slight study effect can be observed for all three algorithms, indicating minor variations in segmentation results across different studies. However, this effect is considerably weaker than the influence of magnetic field strength, which remains the primary source of variability in our Figures. Moreover, assessing the impact of study-related differences is not the focus of this article, especially since the number of subjects from different studies within each age range is insufficient to robustly evaluate this effect. Additional sources of variability such as sex and scanner effects are also considered: please refer to Supplementary Section S4, Fig. S3 and Discussion for the sex effect, and Supplementary Section S5, Figs. S4 and Fig. S5 and Discussion for the scanner effect. Please refer to https://gitlab.com/geodaisics1/ Estimation_of_reference_curves_for_brain _atrophy_and _analysis_of_robustness_to_machine_effects to view those curves for other regions. The visual differences in reference curves as a function of training data indicate that FastSurfer is not robust to magnetic field intensity.

**Measure of errors between curves**

**Fig. 4:**
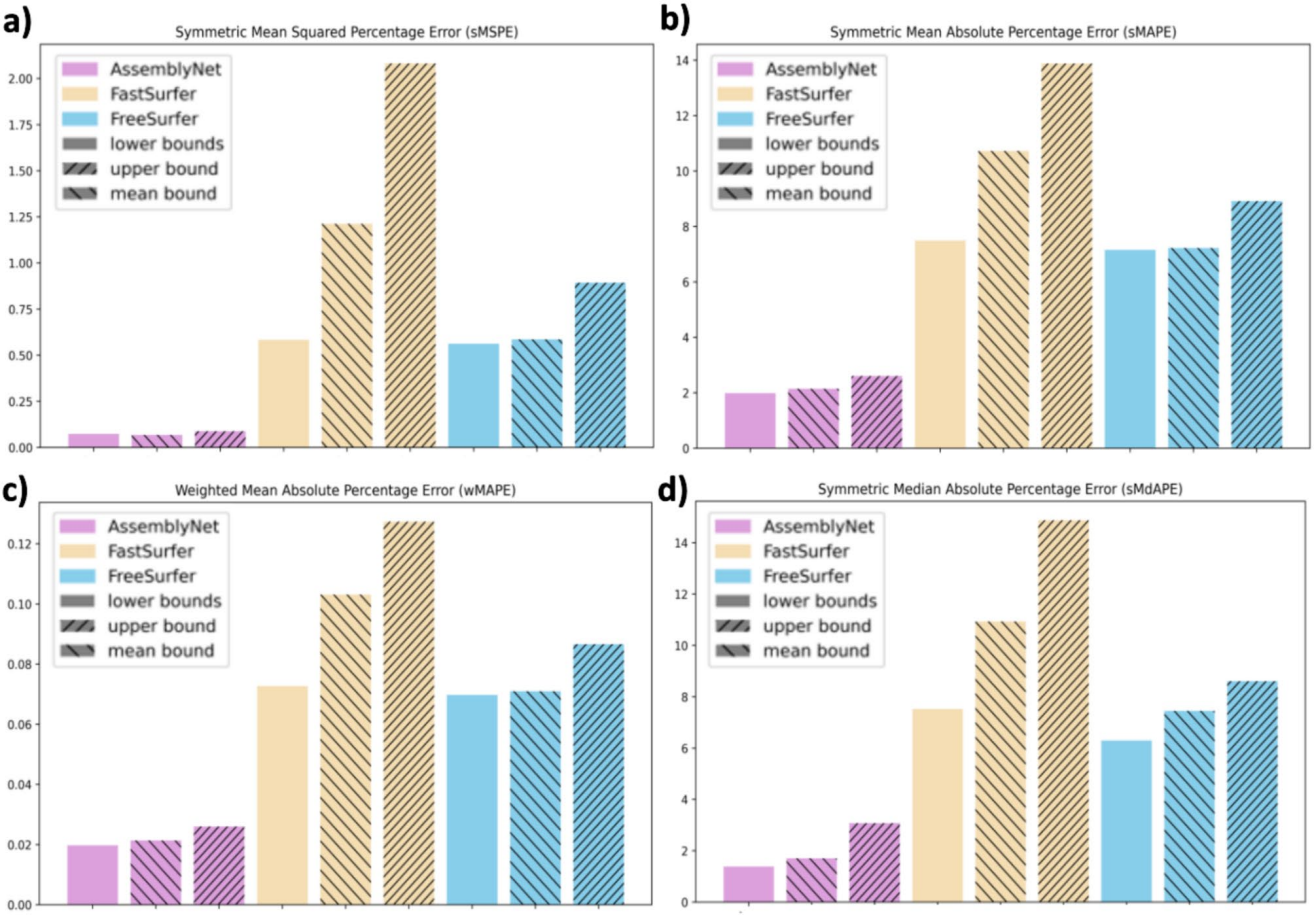
Values of different error metrics: (**a**) sMSPE (symmetric Mean Squared Percentage Error), (**b**) sMAPE (Symmetric Mean Absolute Percentage Error), (**c**) wMAPE (Weighted Mean Absolute Percentage Error), and (**d**) sMdAPE (Symmetric Median Absolute Percentage Error); for the three algorithms (AssemblyNet, FastSurfer, and FreeSurfer). The errors were calculated between the reference curves constructed with data from 1.5T MRI scans and the reference curves predicted using data from 3T MRI scans. AssemblyNet consistently shows lower error metric values compared to FastSurfer and FreeSurfer for all bounds and metrics.

In this section, we assess the performance of segmentation algorithms by directly comparing reference curves calculated with data from 3T MRI scans to curves predicted using data from 1.5T MRI scans (Fig. 4). This approach enables us to evaluate how each algorithm performs when applied across datasets with differing MRI field strengths, reflecting the variability introduced by different MRI field strengths. The robustness of the segmentation algorithms is assessed based on their ability to maintain reference values despite changes in the magnetic field strength.

AssemblyNet achieves the lowest sMSPE for all bounds. Since sMSPE emphasizes larger errors, the smaller values for AssemblyNet indicate that its predictions are consistently closer to the reference curves with fewer large errors. AssemblyNet consistently on sMAPE values outperforms FastSurfer and FreeSurfer across all bounds with lower sMAPE values. A lower sMAPE indicates that AssemblyNet is more robust to magnetic field strength. The lower wMAPE for AssemblyNet indicates that it can maintain high accuracy across different volume sizes, making it more reliable when the data has a wide range of volumes. AssemblyNet achieves the lowest sMdAPE for all bounds. The results for the MASE metric are available in Supplementary Section S6, Fig. S6, and will be discussed in the Discussion section. In conclusion, AssemblyNet consistently outperforms FastSurfer and FreeSurfer in terms of robustness. AssemblyNet’s superior performance across these metrics, especially sMAPE, sMSPE, and wMAPE, indicates that it is more capable of providing reliable volume predictions, even when faced with the variability introduced by different MRI field strengths.

**Method for identifying biases related to magnetic field strength: bootstrapping**

**Fig. 5:**
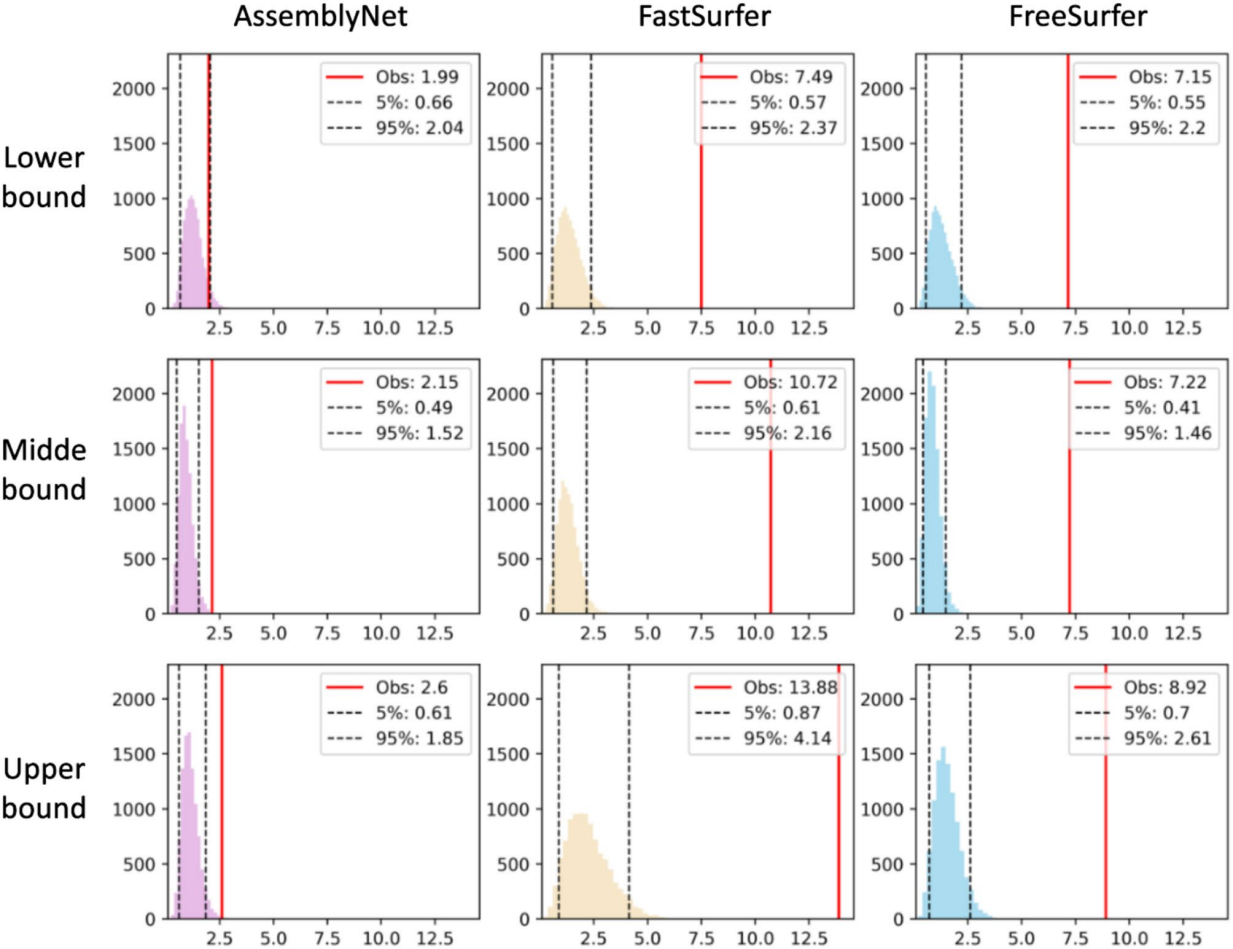
Histograms showing the distribution of sMAPE (symmetric Mean Absolute Percentage Error) values across 10,000 bootstrap iterations with randomized field strength labels. Columns correspond to segmentation algorithms (AssemblyNet, FastSurfer and FreeSurfer), while rows represent boundary conditions (lower, mean and upper bounds). The red line indicates the observed/true label value (“Obs”), i.e., the error metric calculated between the 1.5T and 3T reference curves using the true field strength labels, and the black lines mark the 5th and 95th percentiles. The histogram represents the distribution (y) of metric values (x) obtained from 10,000 bootstrap iterations with randomized field strength labels. This distribution estimates the range of metric variability expected by chance. If the observed value (red line, the true metric value) falls outside the 5th-95th percentile of the bootstrap distribution, it indicates a statistically significant bias likely driven by magnetic field strength rather than random variability. AssemblyNet exhibits fewer true-label values outside the 5th-95th percentiles compared to FastSurfer and FreeSurfer, indicating it is less biased by differences related to magnetic field strength.

The evaluation of bias related to the magnetic field strength, comparing 1.5T and 3T MRI reference curves using bootstrapping, reveals distinct patterns across the three algorithms (Fig. 5 illustrates sMAPE values; for other metrics and views, please refer to Supplementary Section S7, Fig. S7 (sMdAPE), Fig. S8 (sMSPE) and Fig. S9 (wMAPE)).

For AssemblyNet, the true-label values of the sMAPE, sMdAPE and wMAPE fall within the bootstrapped bounds for the lower bound but are slightly outside the 95th percentile for the mean and upper bound indicating a slight magnetic field-related bias. Conversely, sMSPE values for AssemblyNet consistently fall within the bootstrapped bounds across all bounds. In contrast, FastSurfer shows significant deviations for all metrics across configurations, indicating a pronounced bias likely driven by magnetic field effects. Similar trends are observed for sMdAPE and wMAPE. The substantial distances highlight that FastSurfer metrics are heavily influenced by magnetic field strength. FreeSurfer displays similar trends, with marked deviations across metrics, again reflecting strong magnetic field-related effects. In summary, while AssemblyNet exhibits minor deviations, FastSurfer and FreeSurfer demonstrate significant biases across all metrics. These findings suggest that FastSurfer and FreeSurfer are more sensitive to magnetic field differences, while AssemblyNet provides relatively stable results.

### Atrophy assessment

#### Hippocampal atrophy sensitivity analysis

Table 3 shows the percentage of subjects with left/right hippocampal atrophy. For the control group, the percentages of subjects showing hippocampal atrophy according to AssemblyNet are notably consistent across all reference curves. In contrast, FastSurfer exhibits variability. FreeSurfer also shows some variability, though it is more stable compared to FastSurfer. For the pathological group, the percentage of Alzheimer’s patients with hippocampal atrophy is very stable with AssemblyNet, while FastSurfer shows more variability than FreeSurfer. There is a significant difference (McNemar test, p < 0.05)(R version 4.5.0) in the atrophy percentage between AssemblyNet and FreeSurfer, and between FastSurfer and FreeSurfer, across all models.

**Table 3:** Percentage of control and AD subjects showing left/right hippocampal atrophy (for each cell: value1: left/value2: right), according to algorithms (columns) and reference curves used (rows) (concave smoothing). Controls: controls subjects; AD: Alzheimer’s patients. For both groups, AssemblyNet shows a stable percentage of subjects with hippocampal atrophy, while FastSurfer exhibits more variability than FreeSurfer.

**Reference curves**	**Controls**			**AD patients**		
	**AN**	**Fast**	**Free**	**AN**	**Fast**	**Free**
1.5T+3T data	11.76/8.24	12.94/12.55	9.41/9.02	77.17/67.58	84.47/80.82	81.74/76.26
1.5T data	10.59/8.24	7.45/7.06	5.10/4.31	77.17/68.04	80.37/73.52	70.78/67.58
3T data	12.6/8.24	22.75/19.61	12.16/12.94	78.54/67.12	89.50/85.84	86.30/79.91

**Table 4:** Classification scores related to the left/right hippocampus (for each cell: value1: left/value2: right), with rows corresponding to models and columns to segmentation algorithms.

**Metrics**	**References**	**AssemblyNet**	**FastSurfer**	**FreeSurfer**
Sensitivity	1.5T and 3T	0.77/0.68	0.85/0.81	0.82/0.76
	1.5T	0.78/0.68	0.81/0.74	0.75/0.71
	3T	0.79/0.68	0.89/0.86	0.87/0.81
Specificity	1.5T and 3T	0.87/0.91	0.86/0.87	0.90/0.91
	1.5T	0.89/0.91	0.92/0.93	0.93/0.93
	3T	0.87/0.91	0.76/0.80	0.87/0.86
Balanced Accuracy	1.5T and 3T	0.82/0.80	0.86/0.84	0.86/0.84
	1.5T	0.83/0.80	0.86/0.84	0.84/0.82
	3T	0.83/0.79	0.83/0.83	0.87/0.84
Area under the ROC curve	1.5T and 3T	0.85/0.81	0.90/0.88	0.88/0.86
	1.5T	0.85/0.81	0.88/0.85	0.85/0.83
	3T	0.86/0.81	0.91/0.89	0.91/0.87

Table 4 provides the classification performance scores for the detection of left hippocampal atrophy across segmentation algorithms and reference curves. Supplementary Section S8, Fig. S10 shows ROC curves for the left hippocampus. Although AssemblyNet shows slightly lower classification metrics than FastSurfer and FreeSurfer, it consistently demonstrates greater stability across all reference models. In contrast, FastSurfer and FreeSurfer exhibit more variability, with performance scores fluctuating significantly depending on magnetic field strength. For example, regarding specificity, AssemblyNet ranges between 0.87 and 0.91 (considering both left and right hippocampus and all models), while FastSurfer ranges from 0.76 to 0.93, and FreeSurfer from 0.86 to 0.93. This supports the earlier observation that AssemblyNet is more robust to acquisition differences compared to the other segmentation tools.

#### HAVAs results

Supplementary Section S9, Fig. S11 presents the reference curves for the hippocampus, amygdala, and inferior lateral ventricle, across segmentation algorithms. A sensitivity to magnetic field strength is observed for FastSurfer and FreeSurfer, particularly for the hippocampus and amygdala, as indicated by the distribution of colored points according to field strength. However, as shown in Supplementary Section S9, Fig. S12, the HAVAs score appears to reduce this sensitivity to magnetic field strength, likely due to the two-step z-score normalization applied during its computation.

Table 5 presents the percentages of HAVAs scores considered pathological (i.e., scores falling below the 5th percentile of the reference curve) across CN subjects and AD patients, for each model and segmentation algorithms. In CN subjects, AssemblyNet yields overall lower atrophy percentages compared to FastSurfer and FreeSurfer (with a maximum value of 21.96% for AssemblyNet, versus 40.39% for FastSurfer and 33.73% for FreeSurfer). Moreover, AssemblyNet shows a narrower range of atrophy percentages (5.10% to 14.12%), while FastSurfer and FreeSurfer exhibit wider ranges (16.08% to 40.39% and 14.51% to 33.73%, respectively). In AD patients, the percentage of pathological scores is higher, as expected. AssemblyNet again shows slightly lower values (ranging from 82.19% to 94.98%) compared to FastSurfer (87.21% to 97.71%) and FreeSurfer (84.02% to 96.35%).

**Table 5:** Percentages of abnormally low (i.e., scores falling below the 5th percentile of the reference curve) left/right HAVAs scores (for each cell: value1: left/value2: right) in cognitively normal subjects (CN) and Alzheimer’s disease (AD) patients, for each model considered.

References	**CN**			**AD**		
	AN	FastSurfer	FreeSurfer	AN	FastSurfer	FreeSurfer
1.5T and 3T	9.41/12.55	19.61/18.43	16.86/14.12	89.04/86.30	95.43/92.24	93.15/91.78
1.5T	14.12/21.96	40.39/34.51	33.73/32.94	94.98/93.15	97.71/95.43	96.35/94.98
3T	5.10/9.02	16.08/18.04	14.51/15.69	87.67/82.19	87.21/87.21	84.02/87.67

Table 6 presents the classification scores between AD patients and CN subjects obtained using the HAVAs method, across the different models and algorithms. Supplementary Section S9, Fig. S13 shows the ROC curves for the left HAVAs. When comparing the models derived from 1.5T and 3T data (HAVAs left), FastSurfer and FreeSurfer stand out with higher sensitivity (0.95 and 0.93 respectively), compared to 0.89 for AssemblyNet. However, AssemblyNet achieves both the highest specificity (0.91 vs. 0.80 for FastSurfer and 0.83 for FreeSurfer) and the highest balanced accuracy (0.90 vs. 0.88 for FastSurfer and FreeSurfer). Expanding the analysis to all three models (1.5T-only, 3T-only, and combined)(HAVAs left), we observe that the specificity of FastSurfer and FreeSurfer drops significantly compared to AssemblyNet, with minimum values of 0.60 and 0.66, respectively, versus 0.86 for AssemblyNet. These results suggest that FastSurfer, due to its higher sensitivity, may be more effective at detecting Alzheimer’s cases. However, AssemblyNet, with its superior specificity and balanced accuracy, provides a better trade-off between correctly identifying both AD patients and healthy controls.

**Table 6:** Classification scores obtained with the left/right HAVAs scores (for each cell: value1: left/value2: right), organized by model (rows) and segmentation algorithm (columns).

**Metrics**	**References**	**AssemblyNet**	**FastSurfer**	**FreeSurfer**
Sensitivity	1.5T and 3T	0.89/0.86	0.95/0.92	0.93/0.92
	1.5T	0.95/0.93	0.98/0.95	0.96/0.95
	3T	0.88/0.82	0.87/0.87	0.84/0.88
Specificity	1.5T and 3T	0.91/0.87	0.80/0.82	0.83/0.86
	1.5T	0.86/0.78	0.60/0.65	0.66/0.67
	3T	0.95/0.91	0.84/0.82	0.85/0.84
Balanced Accuracy	1.5T and 3T	0.90/0.87	0.88/0.87	0.88/0.89
	1.5T	0.90/0.86	0.79/0.80	0.81/0.81
	3T	0.91/0.87	0.86/0.85	0.85/0.86
Area under the ROC curve	1.5T and 3T	0.93/0.90	0.94/0.93	0.93/0.94
	1.5T	0.95/0.93	0.92/0.91	0.92/0.92
	3T	0.93/0.89	0.89/0.89	0.88/0.90

#### Atrophy stability analysis

**Longitudinal analysis**

Supplementary Section S10, Fig. S14, Fig. S15 and Fig. S16 shows left hippocampal volumes of control and AD subjects from the MIRIAD dataset, acquired during follow-ups, overlaid with reference curves from the three algorithms (using 1.5T and 3T data). This comparison enabled us to assess the consistency and reliability of each algorithm in detecting atrophy over time. For the healthy subjects, most subjects showed no signs of atrophy across all algorithms, indicating that the algorithms generally agree when there is no pathological change. However, discrepancies were observed in some cases. For instance, subject 230 was consistently atrophied, with important variations specifically in FreeSurfer’s results. Subject 231 showed no atrophy with AssemblyNet, consistent atrophy with FastSurfer, and fluctuating results with FreeSurfer. In the AD subjects, the majority were consistently identified as atrophied across all algorithms, which aligns with the expected progression of AD. However, there were notable exceptions, such as subject 191, where FreeSurfer and FastSurfer detected atrophy consistently, but AssemblyNet did not until the penultimate session. Overall, these findings suggest that while all three algorithms can reliably detect atrophy, there are differences in sensitivity and stability, particularly in borderline.

**Inter-sites analysis**

In this study, we used MRI data from the SRPBS traveling subjects. Brain volumes extracted from scans acquired at different sites were projected onto the reference curves for evaluation. Details of this analysis are provided in the Supplementary Section S11, Fig. S17. Supplementary Section S11, Table S2 summarizes the atrophy assessments obtained for SRPBS subjects using the three segmentation algorithms. Overall, FastSurfer exhibits greater variability across sites compared to AssemblyNet and FreeSurfer, suggesting reduced robustness in multi-site settings.

## Discussion

The objectives of this study were to evaluate the robustness and sensitivity of three MRI segmentation algorithms in constructing reference curves for brain atrophy assessment in neurodegenerative diseases, with a focus on hippocampal volumes. The study aimed to determine which algorithm produces the most stable reference curves under varying magnetic field conditions and which algorithm provides the most consistent detection of hippocampal atrophy, a key marker in Alzheimer’s disease. Evaluating the impact of the machine effect and sensitivity is essential, as variability in MRI acquisition parameters and algorithm performance can compromise the reliability and the conclusion of study of reference curves.

Our results indicate that AssemblyNet is the most robust algorithm, consistently generating stable reference curves with minimal impact from variations in magnetic field strength. This robustness is particularly valuable in industrial settings, where data often come from heterogeneous sources, including varying MRI field strengths and different scanner models. In such contexts, covariates like magnetic field strength may not be correctable. Therefore, segmentation methods that are intrinsically robust to these variations are highly advantageous for large-scale deployment. In contrast, FastSurfer displays significant instability, with notable differences in reference curves depending on the MRI field strength. This lack of stability suggests that FastSurfer may not be the optimal choice when consistency across different imaging environments is required. Additionally, AssemblyNet also provides more consistent estimates of hippocampal atrophy and HAVAs scores compared to the other algorithms, though the atrophy percentages were generally lower.

### Reference modeling: addressing confounding factors including imaging variability

#### Accounting for confounders in normative modeling

Confounding variables such as age, sex, scanners effects and MRI field strength can influence brain volumetric measurements and impact the construction of normative models. Addressing these confounders is essential for building accurate and generalizable lifespan trajectories. A review^5^ found that, among 13 normative modeling studies, 7 included only age as a covariate, 4 included both age and sex. Only one study^55^ accounted for field strength, but without justifying this choice.

In our approach, age is directly modeled through GAMs, while sex-related differences are addressed via ICV normalization^56^^,^^57^^,^^58^^,^^59^. This approach is widely used to compensate for head size differences, a major source of inter-individual variability in brain volume measurements. Without ICV correction, differences in regional volumes may reflect cranial size rather than meaningful biological effects. For instance, part of the apparent sex difference in brain volumes is explained by ICV disparities and applying normalization substantially reduces or eliminates these differences^59^^,^^60^. ICV normalization is particularly important for assessing regional volume changes relative to maximum brain size, which is critical in studies of atrophy^61^. It also improves statistical sensitivity, enabling group comparisons with smaller sample sizes, especially in hippocampal studies^62^. Furthermore, ICV normalization contributes to reducing scanner-related variability, thereby enhancing comparability across MRI platforms^9^. Previous work using AssemblyNet has shown that, without normalization, males consistently exhibit larger brain volumes than females across most structures^20^. However, after ICV normalization, these differences largely disappear, suggesting that explicit inclusion of sex as a covariate may not be necessary. Our findings support this interpretation: without normalization, small sex effects were observed across all algorithms, with males generally showing higher hippocampal volumes. After normalization, these effects became very small for AssemblyNet, while FastSurfer and FreeSurfer exhibited persistent differences (please refer to Supplementary Section S4, Fig. S3).

In summary, our modeling framework accounts for age via GAMs and sex through ICV normalization. Field strength was not included as a formal covariate, but its influence was assessed post hoc. Scanner-related effects are discussed in more detail in Section 4.1.

#### Magnetic field strength and scanner model effects: considerations and limitations

Machine effects are complex and multifactorial, as reported in numerous studies^63^^,^^9^^,^^64^^,^^65^. The variability observed in data can indeed arise from multiple sources, including not only magnetic field strength^66^^,^^67^^,^^63^, but also vendor^64^, model^9^, software version^68^, sequence parameters^8^^,^^65^ or gradient non-linearities^69^. Although some studies report minimal effects of magnetic field strength on volumetric estimates, especially for global measures^66^^,^^63^, others have shown regional differences, particularly in areas with low contrast or complex anatomy^70^. Moreover, Marchewka et al., (2014)^70^ did not observe significant differences in hippocampal or amygdalar volumes between field strengths in epilepsy cohorts, especially when using high-quality standardized protocols such as those from ADNI. However, Marchewka et al., (2014)^70^ also reported magnetic field strength-related differences in gray matter volume in the cerebellum, precentral cortex, and thalamus, regions known to be sensitive to scanner parameters and segmentation challenges. These regional effects are consistent with several previous studies^71^^,^^72^, and segmentation accuracy has been shown to improve at 3T in difficult regions due to better contrast-to-noise ratio^70^. In parallel, scanner model effects are also documented. For example, Yang et al., (2016)^64^ found significant variability across models in over 12% of brain regions using FreeSurfer.

In this work, we chose to focus specifically on the magnetic field strength (1.5T vs. 3T) as a proxy of machine-related variability for several reasons. First, as shown in the Supplementary Section S5, Fig. S5, within a given scanner model (Intera), volumetric differences between 1.5T and 3T scans were greater than those observed between different scanner models operating at the same field strength. This suggests that, in our dataset, magnetic field strength exerts a more dominant effect than vendor or model. Second, scanner models were not evenly distributed across age groups, limiting our ability to disentangle model and vendor effects independently of age (please refer to Supplementary Section S5, Fig. S4). Such an analysis would require well-balanced subgroups across all age bins and scanner types, which is not achievable with the current data. Given these considerations, magnetic field strength was selected as the most interpretable and impactful axis of variability for this robustness study. We acknowledge that more granular investigations of vendor, model, and sequence-related effects would be valuable.

#### The “gold standard” issue

Manual segmentation is traditionally used as the reference, often termed the “gold standard”, for evaluating automated brain segmentation methods. However, this designation is increasingly questioned, as manual labeling suffers from limited reproducibility, with intra-rater agreement frequently falling below 80%. For example, using the BrainCOLOR protocol, intra-rater Dice scores (i.e., between repeated manual segmentations) were estimated at 76.8%^44^. This level of agreement is comparable to the consistency observed between manual and automated segmentations: 75.8% for AssemblyNet using the same protocol, and approximately 80% for FastSurfer (80.19% subcortical, 80.65% cortical) on the Mindboggle-101 dataset^47^. Moreover, Coupe et al., (2020)^44^ reported intra-method Dice scores of 92.8% for AssemblyNet (i.e., between scan and rescan automatic segmentations), again surpassing the manual intra-rater reproducibility. These results suggest that automated methods can be as consistent as human experts. These findings challenge the conventional use of manual segmentation as a gold standard.

Although manual segmentations can serve as a reference for partial evaluation, this approach has important limitations. Both human raters and algorithms may exhibit similar biases, particularly in regions with ill-defined anatomical boundaries. This issue also applies to many automated methods: recent deep learning models such as FastSurfer, SynthSeg, and LOD-Brain are often trained on labels generated by FreeSurfer, thereby inheriting its structural biases, for example with hippocampal segmentation, as shown by Valabregue et al., (2024)^73^. These factors make it inherently difficult to determine how close a segmentation truly approximates the underlying anatomical ground truth.

#### Median percentage volume differences between 1.5T and 3T and data

To compare our volumes between 1.5T and 3T and data, we refer to Lee et al., (2024)^74^, who assessed the reliability of FreeSurfer at 1.5T and 3T. They reported the following median volume differences for the left hippocampus: 3.63 cm³ for 1.5T and 3.70 cm³ for 3T, resulting in a 1.89% volume variation between the two field strengths (non-normalized analysis). For FreeSurfer, the higher variation observed for our study (6.15%) could be attributed to the larger and more diverse dataset of 3730 subjects (from 11 different datasets), as opposed to Lee et al., (2024)^74^’s dataset of 101 patients (from 3 datasets). The larger sample size and the inclusion of multiple datasets likely contributed to the increased variability in segmentation results, particularly for FreeSurfer and FastSurfer.

### Comparing modeling strategies for reference curves construction

#### Mean Absolute Scaled Error (MASE) evaluation

The Mean Absolute Scaled Error (MASE) metric meets all inclusion criteria for our study. However, we observed that its value varied a lot depending on the number of points selected (please refer to Supplementary Section S6, Fig. S6). We hypothesize that this variation is due to the fact that our data follow very regular trends, where small errors can lead to elevated MASE values. This is in contrast to the findings of Liu et al., (2024)^24^, who reported MASE values between 0 and 0.15 when comparing reference curves with different datasets. In our case, we observe much higher MASE values, even when selecting only one point out of eight from our reference curves.

#### Comparison of GAM-based curves with existing literature

We compared our GAM-based reference curves to those published in Coupe et al., (2017)^20^, focusing on hippocampal atrophy percentages in both AD and CN subjects (please refer to Supplementary Section S12, Fig. S18 and Fig. S19). The results Supplementary Section S12, Table S3 show that AssemblyNet, when used with our GAM-based models, yields higher atrophy detection rates in AD patients (77.17% left/67.58% right) compared to the reference curves from Coupe et al., (2017)^20^ (63.01%/59.36%). However, for CN subjects, our GAM curves detect a slightly higher percentage of atrophy (11.76%/8.24%) than those of Coupe et al., (2017)^20^ (6.27%/4.31%). This trend is consistent across FastSurfer and FreeSurfer, where GAM-based curves yield both higher detection in AD and slightly elevated atrophy rates in controls. These findings suggest that while the GAM framework may offer improved sensitivity for detecting pathological deviations, it may also slightly increase atrophy detection in CN individuals. This trade-off is further discussed in Section 4.3.

#### Limitations of reference curves and potential of manifold learning for complex analysis

Reference curves are valuable for region-by-region analysis but face limitations in capturing the complexity of high-dimensional MRI data, which often exhibit correlations between brain regions due to the brain’s closed system. This challenge is further compounded by the lack of tools to effectively evaluate the robustness of these curves, particularly in the context of temporal data. In our case, the dataset includes brain volumes from approximately a hundred regions, adding to the analytical complexity. While reference curves are useful for localized assessments, they may fall short in capturing the global dynamics and intricate interdependencies among brain regions. Manifold learning algorithms offer a promising complementary approach by accounting for the interdependence between variables and revealing complex, non-linear relationships within high-dimensional datasets. These methods provide a more integrated and robust framework, enabling a deeper understanding of the global structure and intricate interactions within brain data^75^.

### Atrophy detection across acquisition conditions

#### Early atrophy detection considerations

Our normative lifespan curves suggest that hippocampal volume decline begins at different ages depending on the segmentation algorithm: around 35 years for FastSurfer, 50 years for FreeSurfer, and approximately 55 years for AssemblyNet. However, prior studies indicate that hippocampal atrophy may begin earlier than these estimates suggest^76^^,^^77^. It is important to note that volume trajectories are influenced not only by segmentation accuracy but also by the modeling strategy used to estimate them. In our case, the use of GAMs likely contributes to delay visible inflection points as earlier work using AssemblyNet on larger datasets has shown divergence between AD and normative hippocampal trajectories before the age of 40^78^, suggesting that AssemblyNet is indeed capable of detecting early pathological changes. Therefore, the later onset of decline observed in our curves likely reflects modeling limitations rather than segmentation constraints.

More fundamentally, it is important to recognize that hippocampal atrophy visible on structural MRI occurs relatively late in the pathological cascade of AD. Alzheimer’s disease is characterized by the abnormal accumulation of proteins, including amyloid plaques and neurofibrillary tangles composed of tau. These anomalies disrupt neuronal function and trigger a cascade of toxic mechanisms. Neurons are progressively damaged, lose their connections, and ultimately die^79^^,^^14^^,^^80^^,^^81^. The ATN framework^82^, recently revisited in 2024^83^, offers a biological staging system based on three biomarker categories: A for amyloid accumulation, T for tau pathology, and N for neurodegeneration. CSF biomarkers, measurable through lumbar puncture, can detect amyloid and tau abnormalities up to 15 years before clinical symptom onset^84^^,^^85^. In contrast, neurodegeneration (N), as measured by structural MRI, reflects macroscopic neuronal loss and thus appears later in the disease process. In this context, although MRI remains a valuable tool for assessing neurodegeneration, it inherently captures late-stage processes. As such, efforts to improve early detection of AD may need to focus more on upstream biomarkers (A and T), while MRI-based approaches like ours are better suited for staging and differential diagnosis once neurodegeneration is established. Recent advances in blood-based diagnostics support this shift: improvements in mass spectrometry and antibody-based detection methods have enabled precise quantification of amyloid-$\beta$ and various forms of tau in plasma, paving the way for faster, cheaper, and more accessible detection strategies^86^.

#### Reasons for robustness to magnetic field strength

MRI field strength (1.5T vs. 3T) impacts tissue contrast and signal-to-noise ratio, which can significantly influence segmentation accuracy, especially for methods relying heavily on voxel intensity. Previous studies have shown that higher field strength generally improves segmentation accuracy, particularly in challenging anatomical regions due to better contrast-to-noise ratios^70^. Our results consistently show that AssemblyNet is more robust to magnetic field strength variations compared to FastSurfer and FreeSurfer. This robustness can be attributed to several key aspects of its segmentation architecture and design.

First, AssemblyNet relies on a multiscale 3D deep learning framework composed of 250 U-Net models distributed across two levels of resolution. The first level performs coarse segmentation on overlapping 3D regions (2x2x2 mm3), each independently processed by a dedicated U-Net. A majority voting scheme aggregates the overlapping outputs, effectively introducing spatial redundancy that enhances robustness to contrast variability. The overlap of at least 50% between adjacent regions reinforces this effect by ensuring that the same anatomical region is processed by multiple models. Then, in a second stage, the coarse segmentation is refined using a finer resolution (1x1x1 mm3), improving anatomical boundary delineation. This multi-resolution approach substantially increases the number of learnable parameters (by nearly a factor of ten) allowing more precise modeling of tissue boundaries. According to Coupe et al., (2019)^87^, this two-stage cascade was specifically designed to improve robustness. In addition, the model incorporates a novel nearest-neighbor transfer learning scheme: the weights from a trained U-Net are propagated to adjacent U-Nets processing overlapping regions. This design enables local anatomical knowledge to be shared across the network, which likely further contributes to stable performance across diverse imaging conditions.

In contrast, FastSurfer uses a 2D neural network that segments each slice independently. While this architecture is computationally efficient, it does not exploit the 3D context across slices. As a result, it is more susceptible to slice-wise intensity variations. This likely contributes to its greater sensitivity to field strength variability.

FreeSurfer, on the other hand, employs atlas-based and intensity-driven methods. Subcortical segmentation is based on voxel-wise probabilistic atlases and intensity priors^88^^,^^89^, while cortical surfaces are reconstructed through sulcal and gyral pattern extraction using mesh smoothing and spherical registration^90^^,^^91^. This reliance on local contrast and intensity features makes FreeSurfer vulnerable to variability in MRI contrast and signal quality (contrast-to-noise ratio). This limitation also affects SPM, which, like FreeSurfer, relies on strong anatomical priors (SPM uses voxel intensities and tissue probability maps)^92^.

In summary, the superior robustness of AssemblyNet likely stems from its 3D patch-based architecture, redundant spatial encoding, and multi-resolution refinement, which provide enhanced tolerance to contrast variability. In contrast, FastSurfer’s 2D architecture and FreeSurfer’s reliance on contrast-dependent features make them more susceptible to changes in magnetic field strength.

#### Sensitivity of algorithms to hippocampal atrophy

We assessed the sensitivity of AssemblyNet, FastSurfer, and FreeSurfer in detecting pathological changes, such as hippocampal atrophy. All three segmentation algorithms of our study have demonstrated their capability to detect pathological variations in brain imaging. FreeSurfer has been validated by Fellhauer et al., (2015)^93^, showing its effectiveness in identifying increased brain atrophy in conditions such as AD and mild cognitive impairment. Both FastSurfer and FreeSurfer can detect differences in cortical areas associated with disease progression^94^^,^^47^. AssemblyNet further extends this ability, as demonstrated by Coupe et al., (2022)^32^, where AssemblyNet, combined with classification-based approaches using lifespan models, showed very accurate detection of AD (AUC $\ge$ 94%) compared to control subjects. Additionally, it was able to accurately discriminate between progressive MCI and stable MCI (AUC = 78%) during a 3-year follow-up.

Our analysis shows that AssemblyNet not only provides stable reference curves but also offers consistent estimates of hippocampal atrophy. In contrast, FastSurfer and FreeSurfer showed greater variability in atrophy detection. This variability could lead to important differences in the interpretation of atrophy, highlighting the need for careful consideration when selecting segmentation algorithms for atrophy assessment.

Although AssemblyNet tends to report lower atrophy percentages, these results are in line with De et al., (1997)^95^, but only for the left hippocampus. This study uses the Scheltens scale as a method of determining whether a hippocampus is atrophied: it’s a visual scale. This study includes mild AD patients and patients with moderate to severe AD. The study found frequencies of hippocampal atrophy ranged from 78% in the mild AD patients group to 96% in the advanced AD group. In comparison, the analysis using AssemblyNet (Table 3), using reference curves with both 1.5T and 3T data, revealed an atrophy rate of 77% (left) and 68% (right) (FastSurfer: 84%/81%; FreeSurfer: 82%/76%).

Besides, for all algorithms, there is a disparity in percentages between the left and right hippocampus. This aligns with multiple studies^96^^,^^97^^,^^98^^,^^99^ demonstrating that AD is associated with greater atrophy in the left hippocampus compared to the right.

In addition, De et al., (1997)^95^ found hippocampal atrophy in 15% of the normal elderly group between 60-75 years of age. In our case, the analysis revealed the following percentages of hippocampal atrophy in the control group: AssemblyNet detected 12% atrophy in the left hippocampus and 8% in the right. FastSurfer identified 13% atrophy in both the left and right hippocampus, while FreeSurfer showed 9% atrophy for both sides (MIRIAD: 22 controls; 69 years+/- 16).

We found two other interesting studies on this subject, but neither can be used for comparative analysis as they both use FreeSurfer as their segmentation algorithm. First, in the study by Byun et al^100^, 77.9% of the 163 AD subjects from ADNI are considered to have hippocampal atrophy. Then, in the study Lowe et al^101^, 76% of the 92 AD subjects from ADNI are considered to have hippocampal atrophy.

#### Sensitivity with HAVAs method

As demonstrated by Coupe et al., (2022)^32^, the HAVAs methods offers superior classification performance compared to individual brain regions taken independently, reinforcing its relevance for distinguishing AD patients from CN subjects. When comparing our results to those reported in Coupe et al., (2022)^32^, we observe higher balanced accuracy scores. Specifically, Coupe et al., (2022)^32^ reported a balanced accuracy of 0.81, whereas our current study achieved 0.90 with AssemblyNet, and 0.88 with both FastSurfer and FreeSurfer using reference curves built from combined 1.5T and 3T data. Although the HAVAs method and the segmentation algorithm are the same, several factors explain this discrepancy: the test datasets differ (AIBL in their studies vs. ADNI in ours), the reference models are not the same (GAMs vs. hybrid models), and the training datasets also vary.

#### Trade-off between sensitivity and robustness: bias-variance balance

Our results highlight a classic bias-variance trade-off across segmentation algorithms when applied to AD detection. FastSurfer and FreeSurfer demonstrate higher sensitivity, making them more effective at detecting hippocampal atrophy. This is evidenced in both the direct hippocampal atrophy analysis and the HAVAs-based classification results. However, this increased sensitivity comes at the cost of greater variability and reduced robustness, particularly with respect to acquisition differences. In CN subjects, FastSurfer and FreeSurfer showed broader and less consistent ranges of atrophy percentages (up to 40.39% for FastSurfer), whereas AssemblyNet maintained lower and more stable values (maximum of 21.96%). Across all reference models and field strengths, classification metrics fluctuated more for FastSurfer and FreeSurfer, dropping as low as 0.60 and 0.66 (specificity, HAVAs method), while AssemblyNet consistently maintained higher specificity (0.86-0.95) and balanced accuracy (0.90-0.91)(left HAVAs. These results suggest that AssemblyNet favors a lower-variance strategy, yielding slightly lower sensitivity but offering a more robust and stable performance across different magnetic field strength. While it may miss a few pathological cases, it is more reliable for consistently distinguishing AD patients from healthy individuals without overfitting to magnetic field strength-specific noise.

From an industrial standpoint, robustness is often more desirable than maximal sensitivity. In fact, in a real-world setting, MRI data come from a wide range of machines, models, and sequences. Correction methods such as ComBat^102^ are not always applicable due to insufficient sample size. These harmonization techniques typically require large and balanced datasets, often 20-30 subjects per scanner, per sequence^102^^,^^103^, which are rarely available in industrial settings^7^. Moreover, such harmonization algorithms can introduce additional variability^104^ and may even degrade data quality^105^. Notably, none of the harmonization algorithms evaluated in Gebre et al., (2023)^106^improved intraclass correlation coefficients in longitudinal designs^69^. Thus, a robust algorithm that handles acquisition heterogeneity gracefully is preferable, especially in large-scale screening or multi-site contexts where harmonization is not feasible.

## Conclusion

This study highlights the critical role of algorithm selection in constructing reference curves and assessing brain atrophy in neurodegenerative diseases. We observed that AssemblyNet produces very stable reference curves with respect to magnetic field variations, unlike FastSurfer, which is not robust to this parameter. Significant differences in trends are noted between reference curves constructed with data acquired at 1.5T + 3T, only at 1.5T, and only at 3T. When considering the different reference curves calculated, the percentages of hippocampal atrophy and the HAVAs score in AD patients are more stable with AssemblyNet, though they are lower compared to FastSurfer and FreeSurfer.

In conclusion, AssemblyNet stands out as the most robust and reliable choice, offering stability across varying MRI conditions and consistent atrophy detection. In contrast, FastSurfer and FreeSurfer require further refinement to reduce their sensitivity to magnetic field strength and improve their consistency in atrophy assessment. These findings underscore the importance of segmentation algorithm selection, as the choice of segmentation method can significantly impact the consistency and accuracy of atrophy assessments in neurodegenerative diseases like Alzheimer’s.

## Supplementary Information

Supplementary Information.

## Data Availability

Reference curves associated with this study are available at https://gitlab.com/geodaisics1/Estimation_of_ reference_curves_for_brain_atrophy_and_analysis_of_robustness_to_machine_effects. Reference curves, stored as Excel files, provide the lower, middle, and upper bounds for brain regions, computed for three segmentation algorithms (AssemblyNet, FastSurfer, FreeSurfer) using different MRI field strengths (1.5T, 3T, or both) and smoothing methods (none, concave, convex). Visualizations show the effects of MRI field strength, displaying reference curves with training points color-coded by the field strength. Additional images provide general and consistent visualizations of reference curves, ensuring comparability across algorithms and regions. Concatenated figures summarize multi-algorithm and multi-region comparisons, showing the impact of smoothing and MRI datasets on reference curve generation. These data offer a comprehensive overview of the methods and analyses performed in the study. Links to open-access datasets are also provided. Access to the MRI datasets aggregated here is subject to application procedures individually managed at the discretion of each primary study.
