# Supplementary material for: Estimation of reference curves for brain atrophy and analysis of robustness to machine effects
Source: Sci Rep. 2025 Oct 3;15:34585. doi: 10.1038/s41598-025-18073-z (PMC12494955; doi:10.1038/s41598-025-18073-z)
Supplement: Supplementary file 1 — Supplementary Information. [file 41598_2025_18073_MOESM1_ESM.pdf]

Supplementary Materials

Estimation of reference curves for brain atrophy and analysis of robustness to machine effects

Elodie Piot, Félix Renard, Arnaud Attyé, Alexandre Krainik,
for the Alzheimer's Disease Neuroimaging Initiative
and for the Frontotemporal Lobar Degeneration Neuroimaging Initiative

S1. ERROR METRICS OVERVIEW

This section presents an overview of common error metrics, providing insights for selecting appropriate metrics for our task. We define each metric and discuss its strengths and limitations.

These metrics include the Mean Error (ME), which provides a basic measure of the average deviation, and the Mean Percentage Error (MPE), which expresses the error as a percentage of the reference values. A variant of MPE, called the weighted MPE (wMPE), assigns different weights to different data points based on their importance. The Sum of Absolute Differences (SAD) and Maximum Absolute Error measure the total and largest deviations, respectively, while the Mean Squared Error (MSE) and Root Mean Squared Error (RMSE) focus on the magnitude of errors, with RMSE giving more weight to larger deviations. For errors involving logarithmic transformations, we also consider the Root Mean Squared Logarithmic Error (RMSLE) and the Mean Squared Logarithmic Error (MSLE). The Mean Squared Percentage Error (MSPE) and its symmetric counterpart, the symmetric Mean Squared Percentage Error (sMSPE), measure the squared relative error, with sMSPE balancing the direction of the error. Similar to MPE, the Mean Absolute Error (MAE) provides a straightforward average error, and its scaled version, Mean Absolute Scaled Error (MASE), adjusts for scaling differences between data series. The Mean Absolute Percentage Error (MAPE) is another common metric, with its symmetric version, symmetric Mean Absolute Percentage Error (sMAPE), and the weighted version, weighted Mean Absolute Percentage Error (wMAPE), also considered for comparison. Lastly, the Median Absolute Percentage Error (MdAPE) and symmetric Median Absolute Percentage Error (sMdAPE) offer robust alternatives to MAPE, minimizing the impact of outliers.

In this section, y_i represents the reference values and $\hat{y}_i$ represents the predicted values.

Mean Error (ME): The ME is the average of the prediction errors[1],[2]:

$$ME = \frac{1}{n}\sum_{i=1}^{n}(y_i - \hat{y}_i) \tag{1}$$

Advantages: The ME is easy to interpret, as it directly represents the average magnitude of prediction errors. It is a simple metric to compute and provides insight into the directional bias of a model (whether the model consistently overestimates or underestimates). Additionally, because it is based on an averaging, it is not really sensitive to outliers.

Disadvantages: A key limitation of the ME is that positive and negative errors can cancel each other out, potentially giving the misleading impression of a well-performing model. Moreover, the ME is scale-dependent, meaning it cannot be used to compare performance across tools with different ranges without normalization.

Mean Percentage Error (MPE): The MPE calculates the average percentage error[2]:

$$MPE = \frac{1}{n}\sum_{i=1}^{n}\frac{y_i - \hat{y}_i}{y_i} \times 100 \tag{2}$$

Advantages: The MPE is a simple and interpretable metric. Because it is expressed as a percentage, it is scale-invariant, which makes it especially useful for comparing performance of models with different ranges. Like the ME, it also allows detection of directional bias in the predictions. MPE is only moderately sensitive to outliers, as extreme percentage values can influence the mean, but not as severely as squared-error-based metrics.

Disadvantages: A significant drawback of the MPE is the potential for division by zero, which occurs when the reference values contain zero. This can lead to undefined or infinite results. Additionally, positive and negative percentage errors can offset each other, potentially hiding large errors and giving a misleadingly average percentage error.

weighted MPE (wMPE): The wMPE calculates the MPE and gives greater weight to errors of greater value[3]:

$$wMPE = \frac{\sum_{i=1}^{n}(\hat{y}_i - y_i)}{\sum_{i=1}^{n} y_i} \times 100 \tag{3}$$

Advantages: The wMPE is a scale-invariant metric since it expresses error as a percentage relative to the total magnitude of the reference values. Additionally, by weighting errors according to the reference value, it better reflects the impact of discrepancies on larger values.

Disadvantages: As with MPE, a key limitation is that positive and negative errors can cancel each other out, potentially masking poor performance. Furthermore, the metric is only moderately interpretable, as the global normalization makes it less intuitive than per-instance percentage errors.

Sum of absolute differences (SAD): The SAD is calculated as the total sum of the absolute differences between predicted and true values[4]:

$$SAD = \sum_{i=1}^{n} |y_i - \hat{y}_i| \tag{4}$$

Advantages: The SAD is a straightforward and intuitive metric, as it represents the total amount of error in absolute terms. Unlike metrics such as the Mean Error, errors do not cancel out, since all differences are converted to positive values. This provides a more honest view of overall prediction deviation, especially when over- and underestimation occur simultaneously.

Disadvantages: A primary limitation of SAD is that it is scale-dependent, meaning the magnitude of the value depends on the units and range of the data.

Maximum absolute error (Max-AE): The Max-AE captures the largest single prediction error[5]:

$$Max - AE = \max_i |y_i - \hat{y}_i| \tag{5}$$

Advantages: The Max-AE is a simple and intuitive metric that directly indicates the worst-case error made by the model. It is particularly useful in applications where large individual errors are critical, such as medical diagnosis. Its clarity makes it a useful complement to average-based metrics.

Disadvantages: Like other absolute error metrics, Max-AE is scale-dependent. It is also highly sensitive to outliers, since it focuses solely on the largest deviation, which may not be representative of the model's overall performance.

Mean Squared Error (MSE): The MSE averages the squared differences between predicted and true values, emphasizing larger errors due to squaring[2],[6]:

$$MSE = \frac{1}{n}\sum_{i=1}^{n}(y_i - \hat{y}_i)^2 \tag{6}$$

Advantages: The MSE treats all errors as positive by squaring them, which prevents cancellation of over- and underestimations. Squaring the errors also amplifies larger deviations, making MSE particularly useful in contexts where larger errors should be penalized more heavily.

Disadvantages: The MSE is not scale-invariant, and its value depends on the units of the data squared, which may reduce interpretability. Additionally, it is moderately sensitive to outliers, as the squaring operation disproportionately increases the influence of large errors.

Root Mean Squared Error (RMSE): The RMSE is the square root of Mean Squared Error (MSE), which restores the units of the error metric to match those of the original data[7],[8]:

$$RMSE = \sqrt{MSE} \tag{7}$$

Advantages: The RMSE provides an error measure in the same units as the target variable, making it more interpretable than the MSE. It retains the penalization of larger errors due to the squaring step, while being less extreme than the MSE.

Disadvantages: The RMSE is scale-dependent.

Mean Squared Logarithmic Error (MSLE): The MSLE averages the squared differences between the logarithms of the predicted and true values. It is conceptually similar to the MSE, but both predicted and actual values are transformed using a logarithmic function before comparison[9]:

$$MSLE = \frac{1}{n}\sum_{i=1}^{n}(\log(y_i + 1) - \log(\hat{y}_i + 1))^2 \tag{8}$$

Advantages: The MSLE is scale-invariant (logarithmic scale). This makes it well-suited for comparing values that span several orders of magnitude.

Disadvantages: A key limitation of MSLE is that it cannot handle negative values, as the logarithm is undefined for values below zero. Furthermore, it is less intuitive to interpret than other metrics, since the error is measured in squared log-space rather than in the original units. Additionally, MSLE tends to penalize underestimations more heavily than overestimations[1].

Root Mean Squared Logarithmic Error (RMSLE): The RMSLE is the square root of MSLE[10],[1]:

$$RMSLE = \sqrt{MSLE} \tag{9}$$

Advantages: The RMSLE is less sensitive to large outliers than the standard RMSE, due to the application of the logarithmic transformation. It is also scale-invariant.

Disadvantages: RMSLE cannot handle zero or negative values, since the logarithm is undefined or invalid in these cases. The metric is also less intuitive to interpret, as the error is expressed in transformed log-space rather than the original units. Furthermore, it penalizes underestimations more than overestimations, which may introduce bias.

Mean Squared Percentage Error (MSPE): The MSPE computes the mean squared error in percentage terms by squaring the relative error for each prediction, expressed as a percentage of the reference value[11],[12]:

$$MSPE = \frac{1}{n}\sum_{i=1}^{n}\left(\frac{y_i - \hat{y}_i}{y_i} \times 100\right)^2 \tag{10}$$

Advantages: The MSPE is scale-invariant, since it expresses the error as a squared percentage of the true value.

Disadvantages: Because it squares percentage errors, MSPE is particularly sensitive to large deviations, meaning that a few high-percentage errors can disproportionately impact the final value. Additionally, like other squared-error metrics, it treats over- and under-predictions asymmetrically, potentially introducing bias. It also suffers from the same limitation as other percentage-based metrics: division by small true values can inflate the error dramatically, and the metric cannot handle cases where $y_i = 0$.

symmetric Mean Squared Percentage Error (sMSPE): The sMSPE is a symmetric variant of the Mean Squared Percentage Error (MSPE), designed to treat over- and under-predictions equally[11]:

$$sMSPE = \frac{1}{n} \sum_{i=1}^{n} \left(\frac{2 \times (\hat{y}_i - y_i)}{|y_i + \hat{y}_i|} \times 100 \right)^2 \tag{11}$$

Advantages: The sMSPE is scale-independent, as it expresses the error as a percentage. By using a symmetric formulation, it treats overestimations and underestimations equally. Since it squares the relative errors, it ensures that positive and negative deviations do not cancel out, and it places greater penalty on larger errors compared to metrics like Mean Absolute Percentage Error (MAPE) or Symmetric Mean Absolute Percentage Error (sMAPE). This makes it particularly suitable when large deviations must be heavily penalized.

Disadvantages: The main limitation of sMSPE lies in its moderate interpretability, as the squared symmetric percentage form is less intuitive than absolute percentage errors.

Mean Absolute Error (MAE): The MAE averages the absolute prediction errors (average size of the error) by taking the mean of the absolute differences between predicted and reference values[7],[8]:

$$MAE = \frac{1}{n} \sum_{i=1}^{n} |y_i - \hat{y}_i| \tag{12}$$

Advantages: The MAE is a highly interpretable metric, as it directly reflects the average size of the prediction errors, regardless of their direction. Unlike MSE or RMSE, it does not square the errors, making it less sensitive to outliers.

Disadvantages: The MAE is scale-dependent, meaning its value is expressed in the same unit as the target variable. Additionally, while MAE gives equal weight to all errors, this may be a disadvantage in applications where larger errors should be penalized more heavily.

Mean Absolute Scaled Error (MASE): The MASE scales the Mean Absolute Error (MAE)[11]:

$$MASE = \frac{MAE}{\frac{1}{n-1} \sum_{i=2}^{n} |y_i - y_{i-1}|} \tag{13}$$

Advantages: MASE is scale-independent.

Disadvantages: MASE can become unstable or undefined when the denominator approaches zero, which may occur in datasets with very low variation. Additionally, while it is more robust to outliers than squared-error metrics, it remains moderately sensitive to extreme values, especially if those affect the MAE in the numerator.

Mean Absolute Percentage Error (MAPE): The MAPE is the average absolute percentage error[13]:

$$MAPE = \frac{1}{n} \sum_{i=1}^{n} \left| \frac{y_i - \hat{y}_i}{y_i} \right| \times 100 \tag{14}$$

Advantages: The MAPE is scale-independent, as it expresses error in percentage terms. It is also easy to interpret, as it indicates the average deviation from the reference values in percent, which is often more intuitive than absolute or squared errors.

Disadvantages: A major limitation of MAPE is its instability when true values are close to zero, as this can result in extremely large percentage errors or even division by zero. Additionally, MAPE is not symmetric: it tends to penalize over-predictions and under-predictions unequally, potentially introducing bias[1].

weighted Mean Absolute Percentage Error (wMAPE): The wMAPE weighs each prediction error relative to the scale of the values (a prediction with a higher percentage error will have a stronger impact on the score)[14]:

$$wMAPE = \frac{\sum_{i=1}^{n} |y_i - \hat{y}_i|}{\sum_{i=1}^{n} |y_i|} \tag{15}$$

Advantages: The wMAPE is scale-independent, as it expresses the total absolute error as a percentage of the total reference values. It also avoids issues like division by zero encountered in standard MAPE.

Disadvantages: While useful in many contexts, wMAPE can be moderately interpretable, as it does not represent an average per-instance percentage error but rather a weighted aggregate.

symmetric Mean Absolute Percentage Error (sMAPE) The sMAPE normalizes the error (by the sum of the predicted and reference values). This symmetric formulation avoids the asymmetry present in standard MAPE[15]:

$$sMAPE = \frac{1}{n} \sum_{i=1}^{n} \frac{2 \times |y_i - \hat{y}_i|}{|y_i| + |\hat{y}_i|} \times 100 \tag{16}$$

Advantages: The sMAPE is scale-independent, as it expresses errors in percentage form. Its symmetric structure ensures that over-predictions and under-predictions are treated equally. Additionally, it avoids division by zero by using the sum of absolute values in the denominator.

Disadvantages: While more robust than MAPE, sMAPE remains moderately sensitive to extreme outliers. Its nonlinear normalization can also make interpretation less straightforward compared to more direct error metrics.

Median Absolute Percentage Error (MdAPE): The MdAPE calculates the absolute percentage error for each prediction, and then returns the median of these values, rather than the mean[11]:

$$MdAPE = \text{median}\left(\left|\frac{y_i - \hat{y}_i}{y_i}\right|\right) \times 100 \tag{17}$$

Advantages: The MdAPE is robust to outliers, as it uses the median instead of the mean, which prevents extreme errors from disproportionately influencing the result. It is also scale-independent.

Disadvantages: Like MAPE, MdAPE is not a symmetric metric, meaning that it treats over-predictions and under-predictions differently, which may lead to biased evaluations. Additionally, it can still be unstable when true values are close to zero, due to the percentage formulation.

symmetric Median Absolute Percentage Error (sMdAPE): The sMdAPE computes the median of the symmetric absolute percentage errors, using a denominator that averages the absolute true and predicted values. This formulation ensures balanced treatment of over- and under-predictions[11]:

$$sMdAPE = \text{median}\left(\frac{2 \times |y_i - \hat{y}_i|}{|y_i| + |\hat{y}_i|}\right) \times 100 \tag{18}$$

Advantages: The sMdAPE is a symmetric metric, meaning it treats overestimations and underestimations equally. By using the median, it is also robust to outliers, making it well-suited for datasets that contain occasional large deviations. As a percentage-based metric, it is scale-independent.

Disadvantages: While more balanced and robust than MAPE or MdAPE, the sMdAPE is moderately interpretable, as its formulation, both median-based and normalized, is less intuitive.

Supplementary Table S1 provides a summary of the characteristics of the 18 evaluated metrics with respect to the defined selection criteria.

Group of metrics	Metric name	Easily interpretable	Offsets errors	Scale dependent	Treats over and under prediction differently	Inclusion
	ME	Yes	YES	YES	No	NO
MPE and relative metrics	MPE	Yes	YES	No	No	NO
	wMPE	Moderately	YES	No	No	NO
	SAD	Yes	No	YES	No	NO
	Max-AE	Yes	No	YES	No	NO
MSE and relative metrics	MSE	Moderate	No	YES	No	NO
	RMSE	Moderate	No	YES	No	NO
	RMSLE	Hardly	No	No	YES	NO
MSPE and relative metrics	MSPE	Moderate	No	No	YES	NO
	sMSPE	Moderate	No	No	No	YES
	MSLE	Hardly	No	No	YES	NO
	MAE	Yes	No	YES	No	NO
	MASE	Moderate	No	No	No	YES
MAPE and relative metrics	MAPE	Yes	No	No	YES	NO
	sMAPE	Moderate	No	No	No	YES
	wMAPE	Moderate	No	No	No	YES
MdAPE and relative metrics	MdAPE	Yes	No	No	YES	NO
	sMdAPE	Moderate	No	No	No	YES

Supplementary Table S1: Overview of the 18 error metrics according to evaluation criteria. Metrics: Mean Error (ME), Mean Percentage Error (MPE), weighted MPE (wMPE), Sum of Absolute Differences (SAD), Maximum Absolute Error (Max-AE), Mean Squared Error (MSE), Root Mean Squared Error (RMSE), Root Mean Squared Logarithmic Error (RMSLE), Mean Squared Percentage Error (MSPE), symmetric Mean Squared Percentage Error (sMSPE), Mean Squared Logarithmic Error (MSLE), Mean Absolute Error (MAE), Mean Absolute Scaled Error (MASE), Mean Absolute Percentage Error (MAPE), symmetric Mean Absolute Percentage Error (sMAPE), weighted Mean Absolute Percentage Error (wMAPE), Median Absolute Percentage Error (MdAPE) and symmetric Median Absolute Percentage Error (sMdAPE)

S2. CONSTRAINT EFFECTS

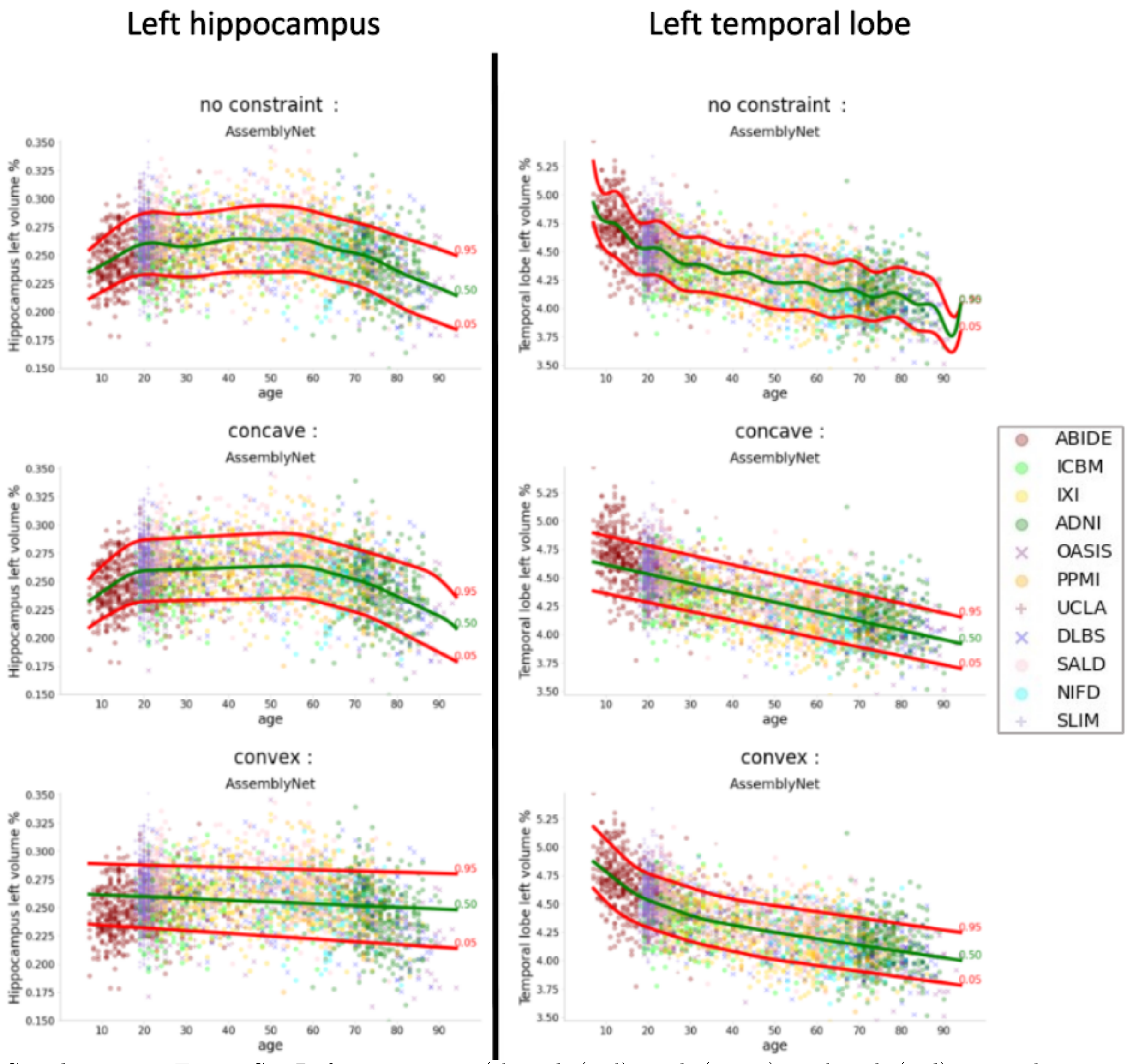

Supplementary Figure S1: Reference curves (the 5th (red), 50th (green), and 95th (red) expectiles were computed) for the left hippocampus (left) and the left temporal lobe (right) (normalized volumes) constructing with no constraint (top), with a concave smoothing (middle) and with a convex smoothing (bottom) (reference curves build with 1.5T and 3T data)

S3. MEDIAN VOLUMES AND MEDIAN PERCENTAGE VOLUME DIFFERENCES BETWEEN 1.5T AND 3T DATA

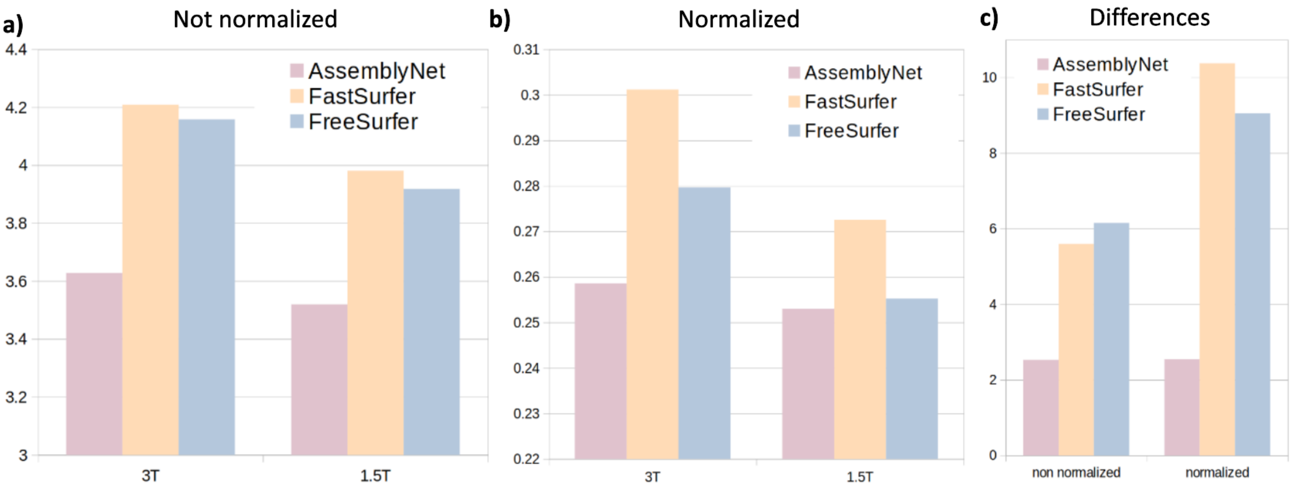

Supplementary Figure S2: Comparison of left hippocampal volume across algorithms and magnetic field strengths: a) non-normalized (mL) and b) normalized (by the intracranial volume) median volumes; and c) median percentage volume differences (volumes of 3730 subjects)

S4. SEX EFFECT ON HIPPOCAMPAL VOLUMES

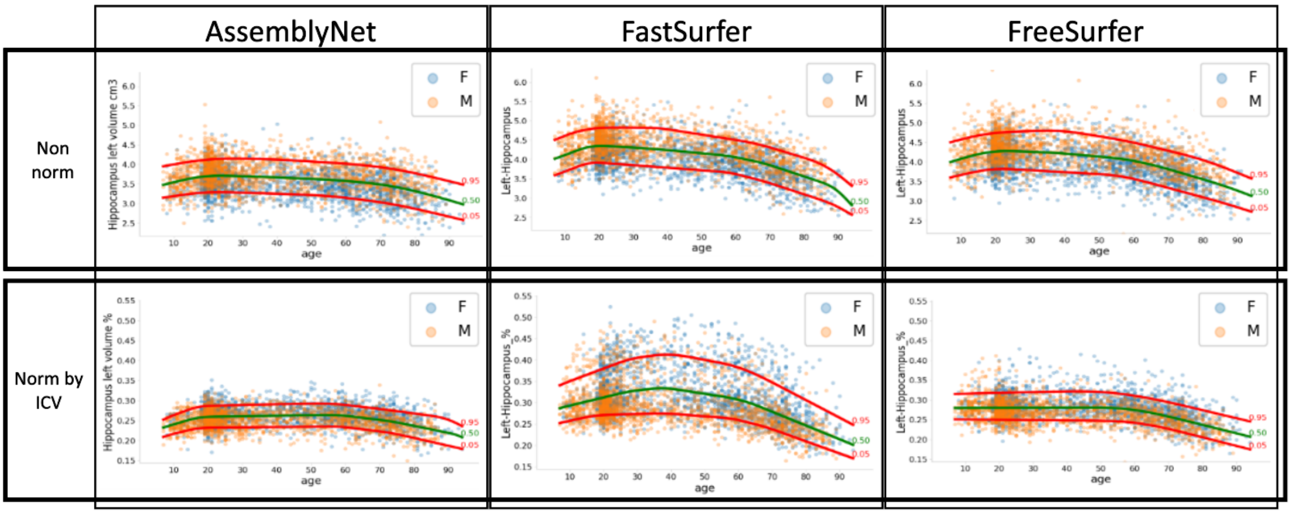

Supplementary Figure S3: Reference curves for the left hippocampus using concave smoothing, computed from combined data acquired at 1.5T and 3T. Top row: non-normalized ("Non norm") volumes. Second row: volumes normalized by intracranial volume ("Norm by ICV"). Data points are colored by gender (F: female, M: male). Each column corresponds to one of the three segmentation algorithms evaluated in the study. Each point represents a single subject. The 5th (red), 50th (green), and 95th (red) expectiles are shown to characterize population variability across the lifespan.

In Supplementary Fig. S3, in the non-normalized volume plots (top row), a slight sex effect is visible across all algorithms, with male subjects tending to have larger hippocampal volumes. However, after normalization (bottom row), this effect diminishes for AssemblyNet (almost complete overlap), and appears even slightly reversed (female points slightly above male ones). For FastSurfer, male and female data points appear more mixed, with female points generally lying above male ones. This effect is more visually pronounced than in AssemblyNet. FreeSurfer shows a similar trend, though less marked than in FastSurfer. As shown in Fig. 3, 3T scans tend to yield higher hippocampal volumes than 1.5T for FastSurfer and FreeSurfer. However, this unexpected inversion is unlikely to be explained by a sex imbalance in 3T data, as the number of females and males scanned at 3T is relatively balanced (1263 females vs. 1289 males).

S5. EFFECTS OF SCANNERS VERSUS EFFECTS OF MAGNETIC FIELDS

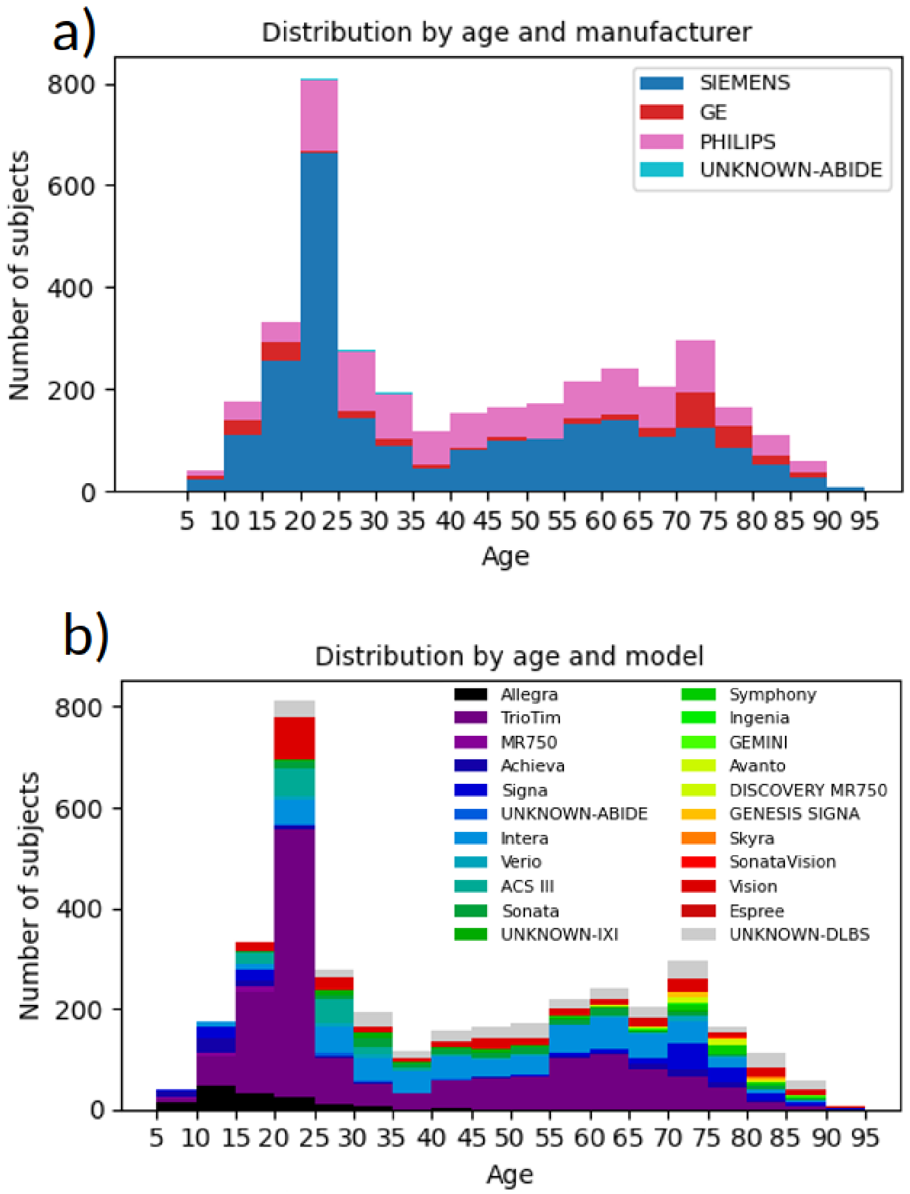

Supplementary Figure S4: a) Distribution of MRI scanner manufacturers by patient age group. b) Distribution of MRI scanner types by patient age group.

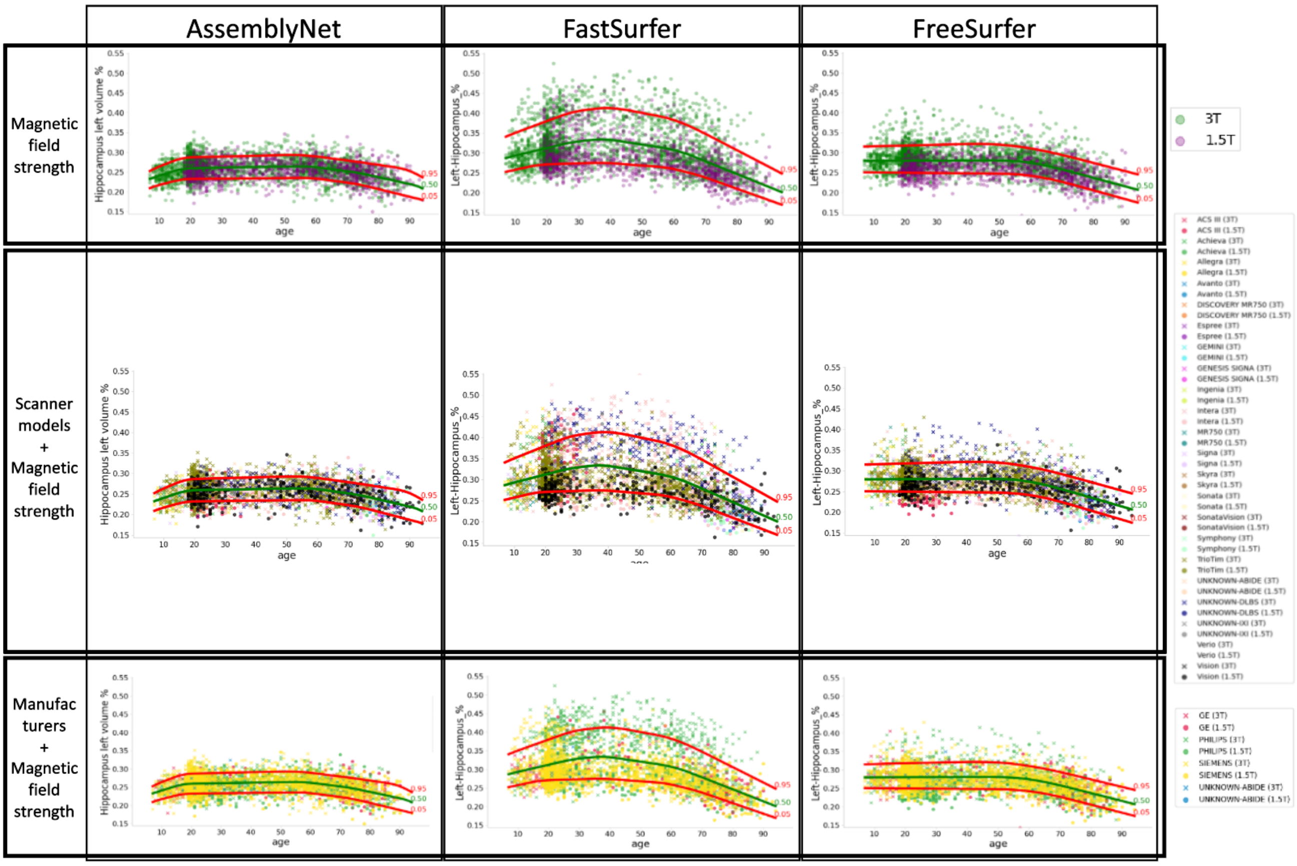

Supplementary Figure S5: Reference curves for the left hippocampus using concave smoothing, computed from combined data acquired at 1.5T and 3T. Top row: data points are colored by magnetic field strength (1.5T vs 3T). Second row: data points are colored by scanner model, with crosses representing 3T and circles representing 1.5T. Third row: data points are colored by manufacturer, again with crosses for 3T and circles for 1.5T. Each column corresponds to one of the three segmentation algorithms evaluated in the study. Each point represents a single subject. The 5th (red), 50th (green), and 95th (red) expectiles are shown to characterize population variability.

In Supplementary Fig. S5, AssemblyNet appears less sensitive to variations in magnetic field strength compared to FastSurfer and FreeSurfer, where data from 3T scanners tend to lie higher than those from 1.5T. Then, in the second row, there appears to be no clear scanner model effect for AssemblyNet and FreeSurfer. However, within the FastSurfer panel, the Intera scanner (pink) at 1.5T (circle) lies noticeably lower than the same model at 3T (cross), supporting the observation that magnetic field strength has a stronger impact than scanner model. In the third row for FastSurfer, Philips scanners (green) show a clear separation: 3T scanners (crosses) appear consistently higher than 1.5T scanners (circles), further suggesting that magnetic field strength exerts a greater influence than manufacturer.

S6. METRIC EVALUATION: MASE

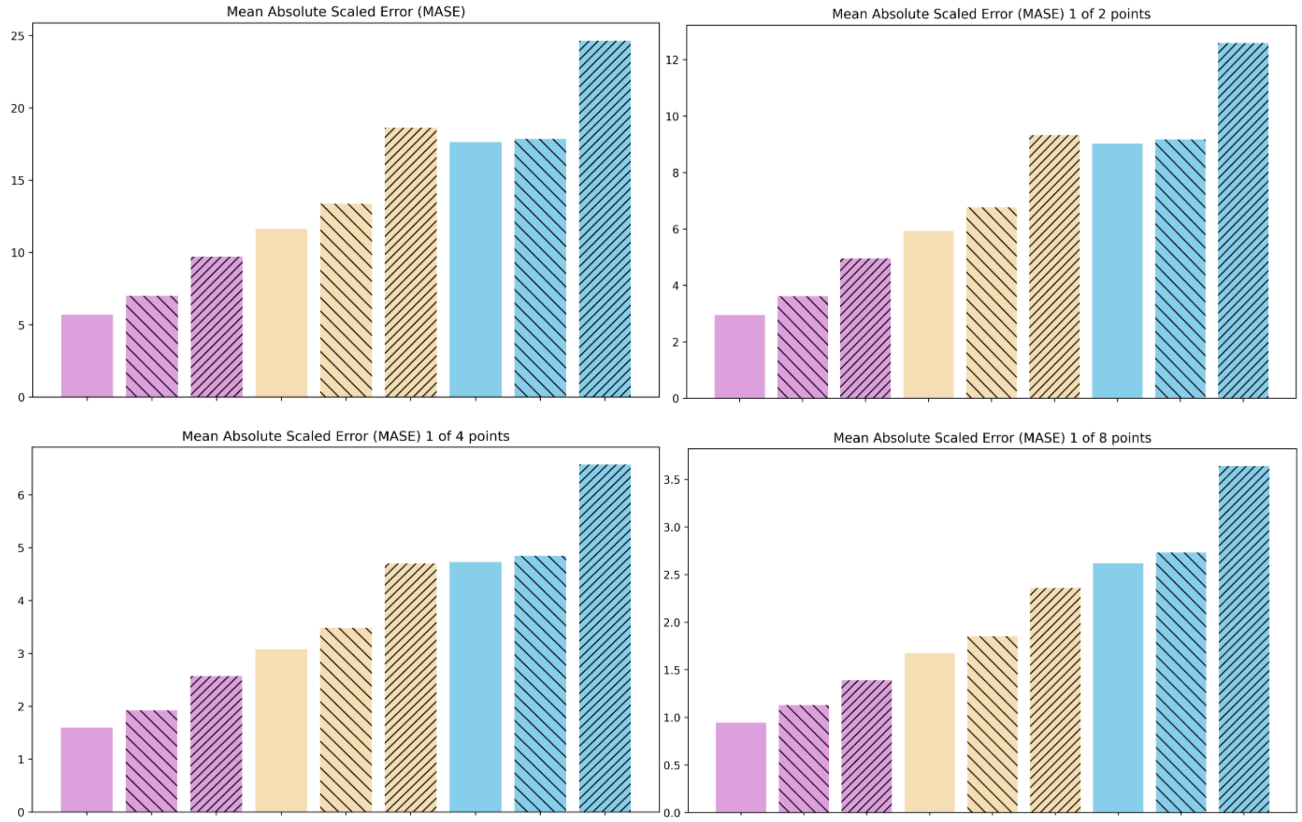

Supplementary Figure S6: MASE (Mean Absolute Scaled Error) values when comparing 1.5T versus 3T for the three algorithms and three bounds. Top left: one point per age (using the entire set of points). Top right: one point every 2 years. Bottom left: one point every 4 years. Bottom right: one point every 8 years

S7. BOOTSTRAP RESULTS

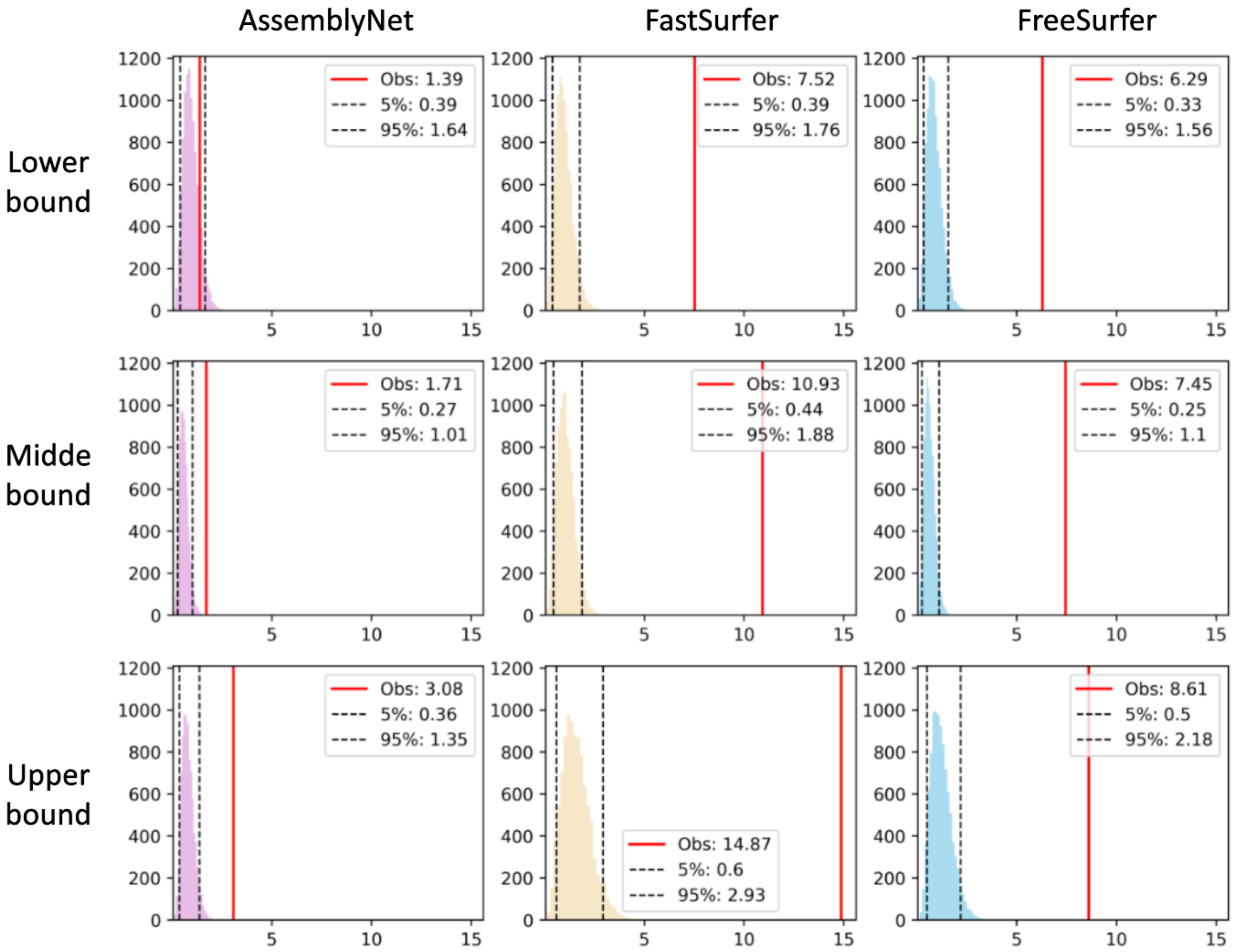

Supplementary Figure S7: Histograms showing the distribution of sMdAPE (symmetric Median Absolute Percentage Error) values across 10,000 bootstrap iterations with randomized field strength labels. Columns correspond to segmentation algorithms (AssemblyNet, FastSurfer and FreeSurfer), while rows represent boundary conditions (lower, mean and upper bounds). The red line indicates the observed/true label value ("Obs"), i.e., the error metric calculated between the 1.5T and 3T reference curves using the true field strength labels, and the black lines mark the 5th and 95th percentiles. The histogram represents the distribution (y) of metric values (x) obtained from 10,000 bootstrap iterations with randomized field strength labels. This distribution estimates the range of metric variability expected by chance. If the observed value (red line, the true metric value) falls outside the 5th-95th percentile of the bootstrap distribution, it indicates a statistically significant bias likely driven by magnetic field strength rather than random variability.

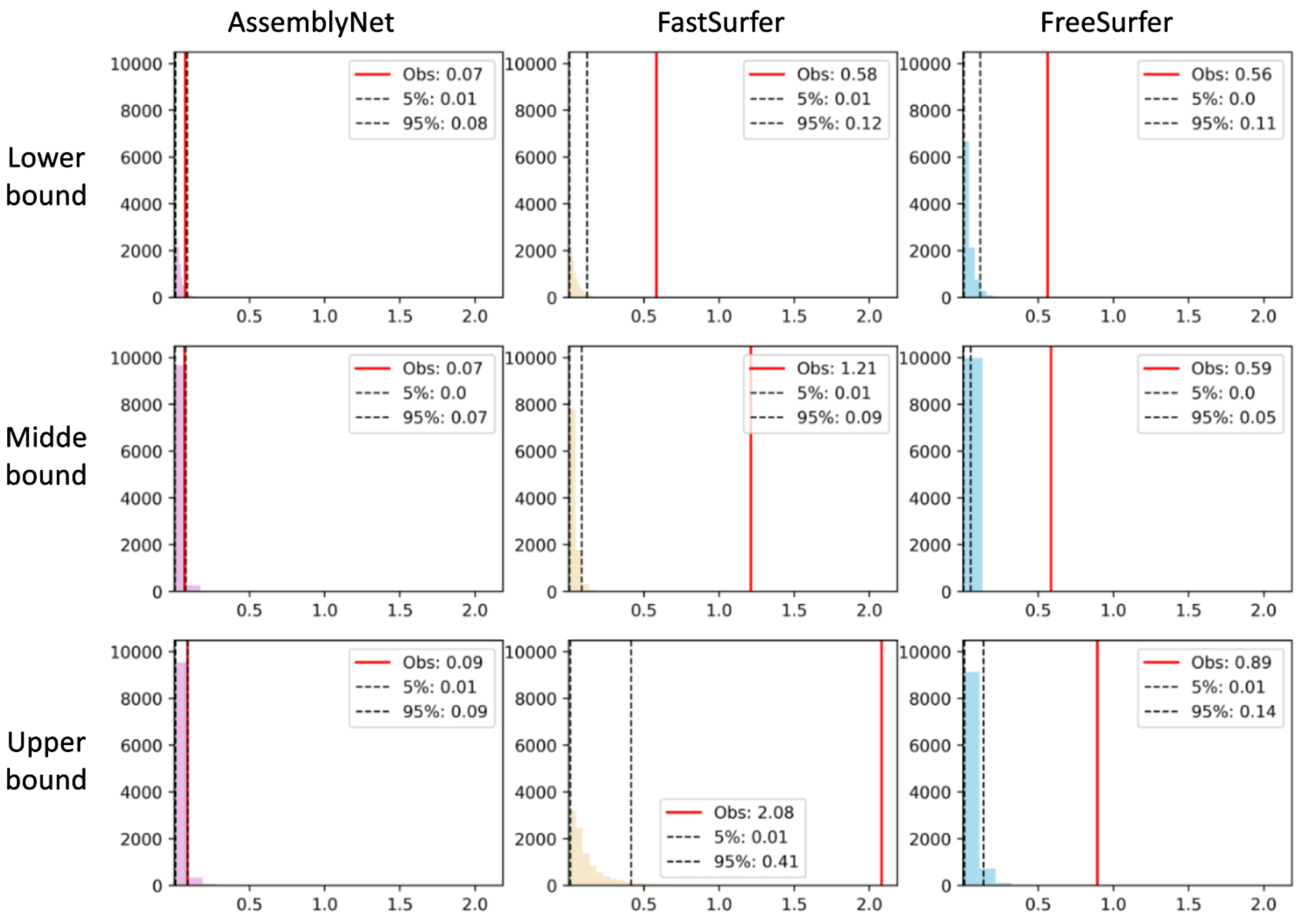

Supplementary Figure S8: Histograms showing the distribution of sMSPE (symmetric Mean Squared Percentage Error) values across 10,000 bootstrap iterations with randomized field strength labels. Columns correspond to segmentation algorithms (AssemblyNet, FastSurfer and FreeSurfer), while rows represent boundary conditions (lower, mean and upper bounds). The red line indicates the observed/true label value (”Obs”), i.e., the error metric calculated between the 1.5T and 3T reference curves using the true field strength labels, and the black lines mark the 5th and 95th percentiles. The histogram represents the distribution (y) of metric values (x) obtained from 10,000 bootstrap iterations with randomized field strength labels. This distribution estimates the range of metric variability expected by chance. If the observed value (red line, the true metric value) falls outside the 5th-95th percentile of the bootstrap distribution, it indicates a statistically significant bias likely driven by magnetic field strength rather than random variability.

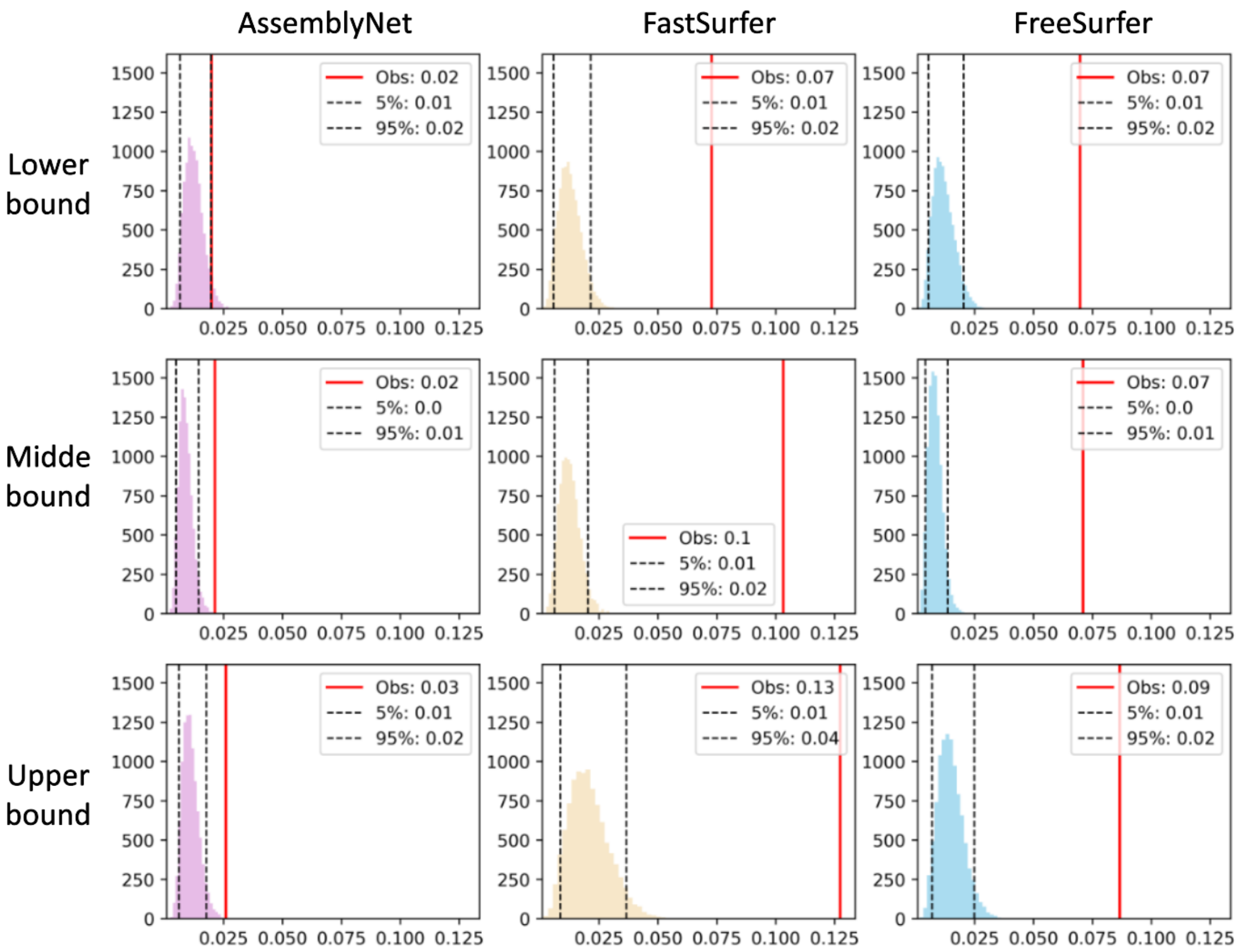

Supplementary Figure S9: Histograms showing the distribution of wMAPE (weighted Mean Absolute Percentage Error) values across 10,000 bootstrap iterations with randomized field strength labels. Columns correspond to segmentation algorithms (AssemblyNet, FastSurfer and FreeSurfer), while rows represent boundary conditions (lower, mean and upper bounds). The red line indicates the observed/true label value ("Obs"), i.e., the error metric calculated between the 1.5T and 3T reference curves using the true field strength labels, and the black lines mark the 5th and 95th percentiles. The histogram represents the distribution (y) of metric values (x) obtained from 10,000 bootstrap iterations with randomized field strength labels. This distribution estimates the range of metric variability expected by chance. If the observed value (red line, the true metric value) falls outside the 5th-95th percentile of the bootstrap distribution, it indicates a statistically significant bias likely driven by magnetic field strength rather than random variability.

S8. ROC CURVES FOR THE LEFT HIPPOCAMPUS

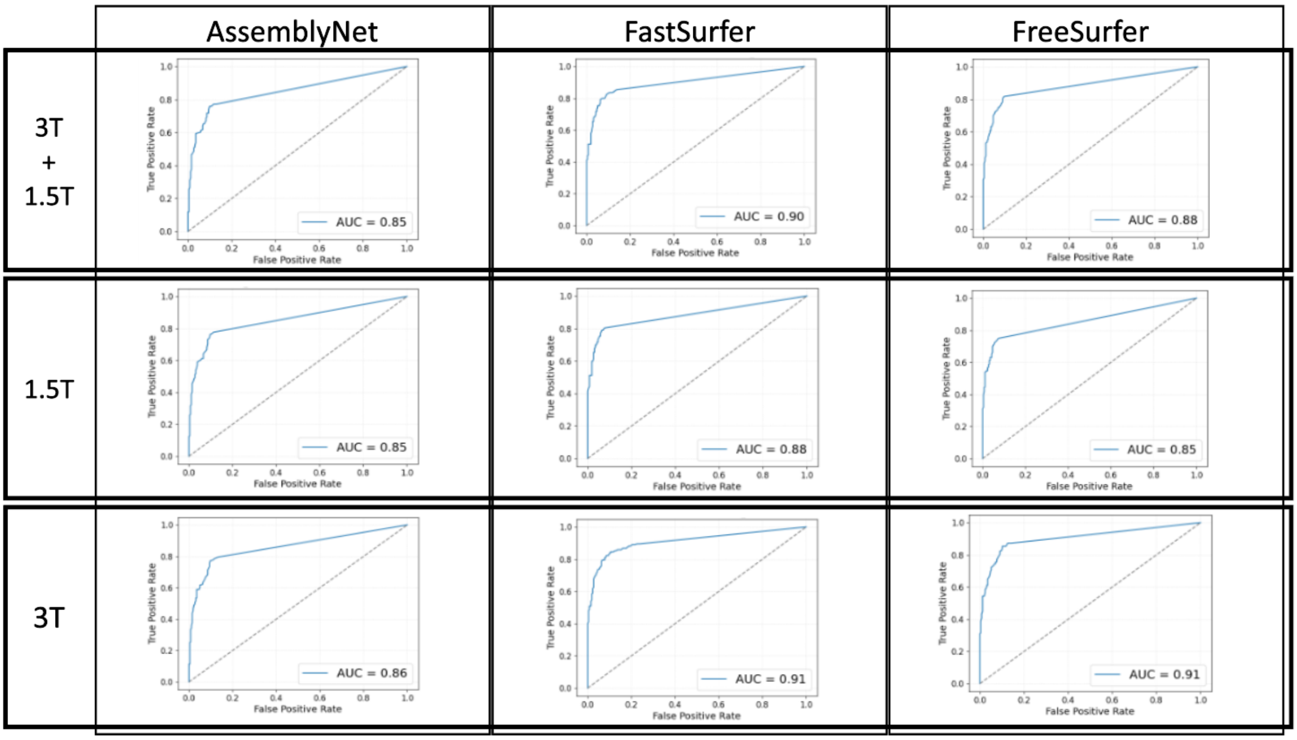

Supplementary Figure S10: ROC curves and AUC for the left hippocampus, computed using three different datasets: combined 1.5T and 3T data (top row), 1.5T data only (middle row), and 3T data only (bottom row). Each column corresponds to one of the three segmentation algorithms evaluated.

S9. REFERENCE CURVES FOR HAVAS

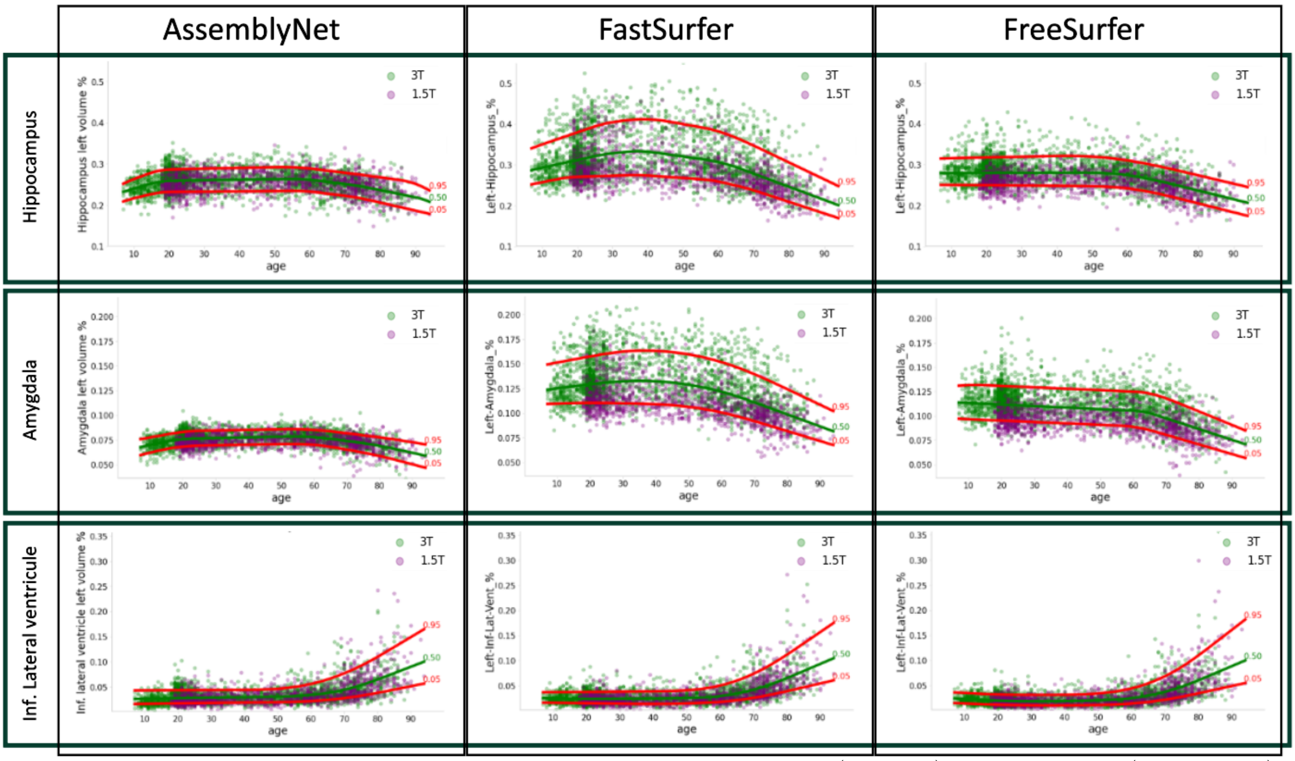

Supplementary Figure S11: Reference curves for the left hippocampus (top row), left amygdala (middle row), and left inferior lateral ventricle (bottom row), across segmentation algorithms (columns). Data points are colored according to magnetic field strength. Each point represents a single subject. The 5th (red), 50th (green), and 95th (red) expectiles are shown to characterize population variability. Curves were computed using data acquired at both 1.5T and 3T.

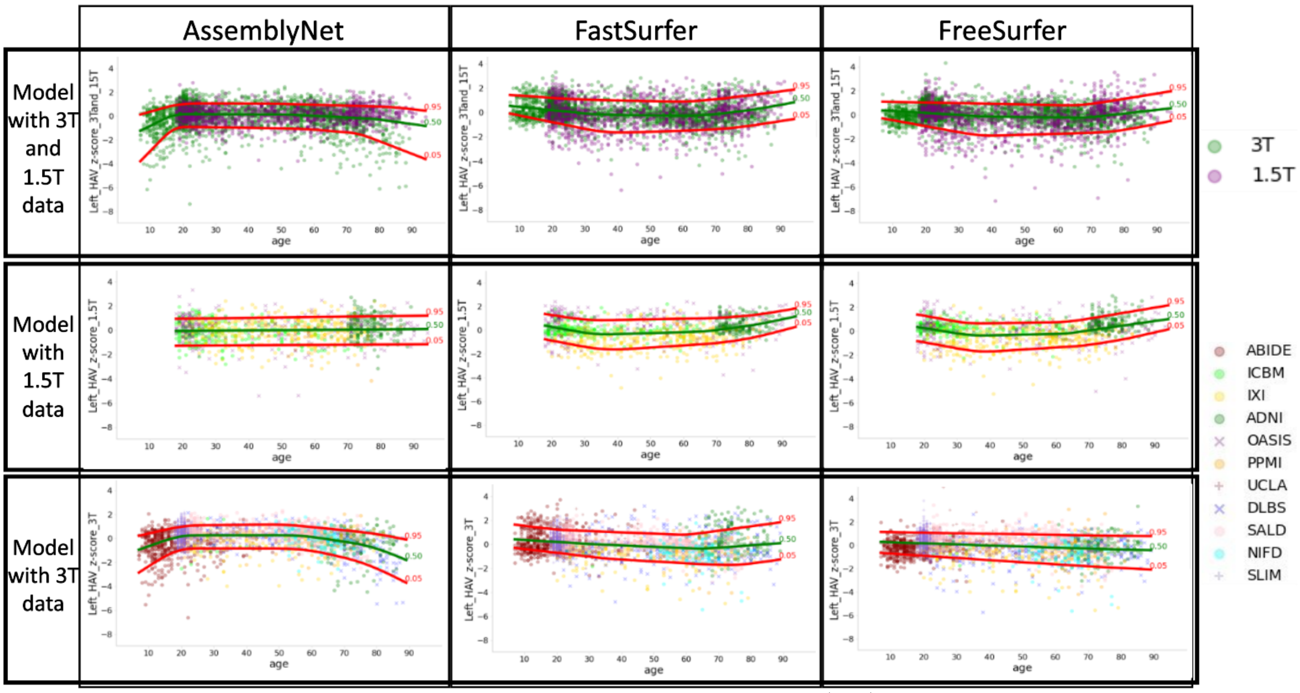

Supplementary Figure S12: Reference curves for the HAVAs score (left), computed using three different datasets: combined 1.5T and 3T data (top row, with points colored by magnetic field strength), 1.5T data only (middle row, with points colored by study), and 3T data only (bottom row, with points colored by study). Each column corresponds to one of the three segmentation algorithms evaluated. Each point represents a single subject. The 5th (red), 50th (green), and 95th (red) expectiles are shown to characterize population variability.

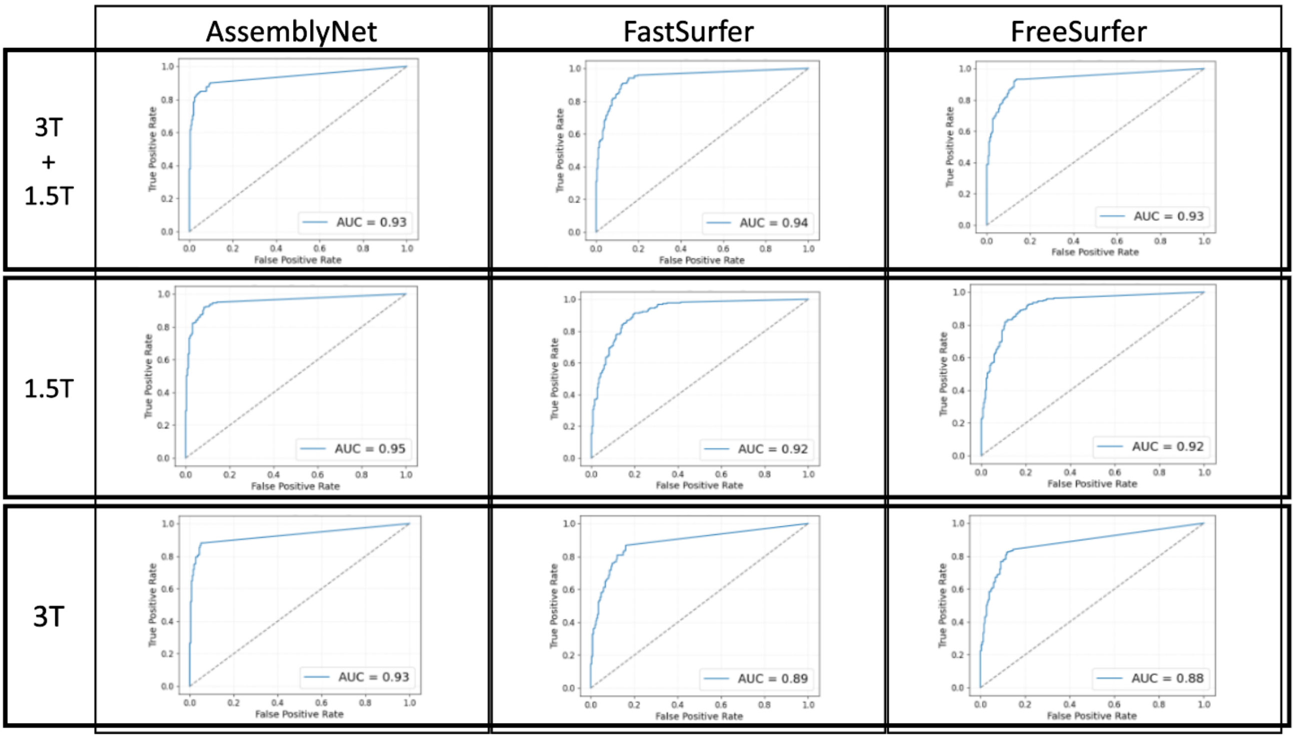

Supplementary Figure S13: ROC curves and AUC for the left HAVAs, computed using three different datasets: combined 1.5T and 3T data (top row), 1.5T data only (middle row), and 3T data only (bottom row). Each column corresponds to one of the three segmentation algorithms evaluated.

S10. EVALUATION OF THE STABILITY OF RESULTS: LONGITUDINAL STUDY

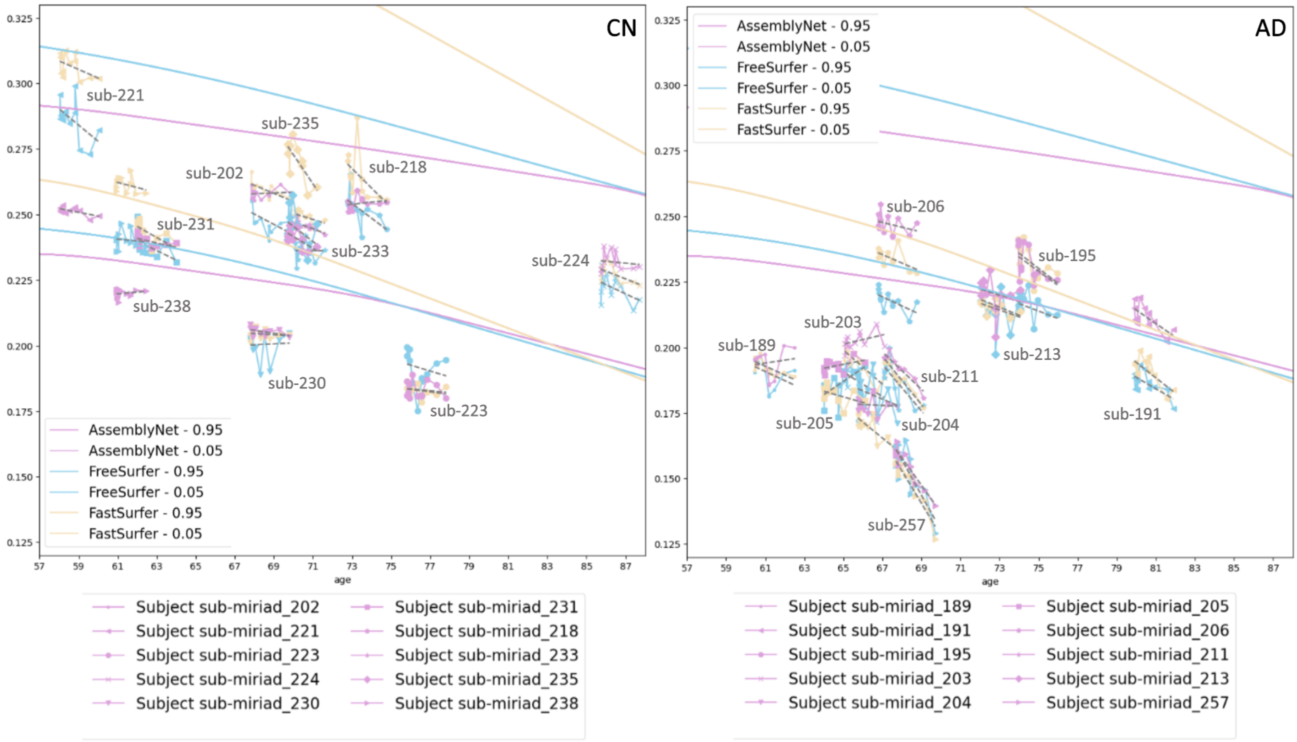

Supplementary Figure S14: Visualization of the evolution of left hippocampal volume for the 20 MIRIAD subjects (left: 10 control subjects; right: 10 Alzheimer's patients (AD)) with the most longitudinal follow-up (reference curves (5th and 95th expectiles) build with 1.5T and 3T data, concave smoothing)

For individual subject views, please refer to the next figures: AD patients in Supplementary Fig. S16 and CN subjects in Supplementary Fig. S15.

Individual CN subjects views:

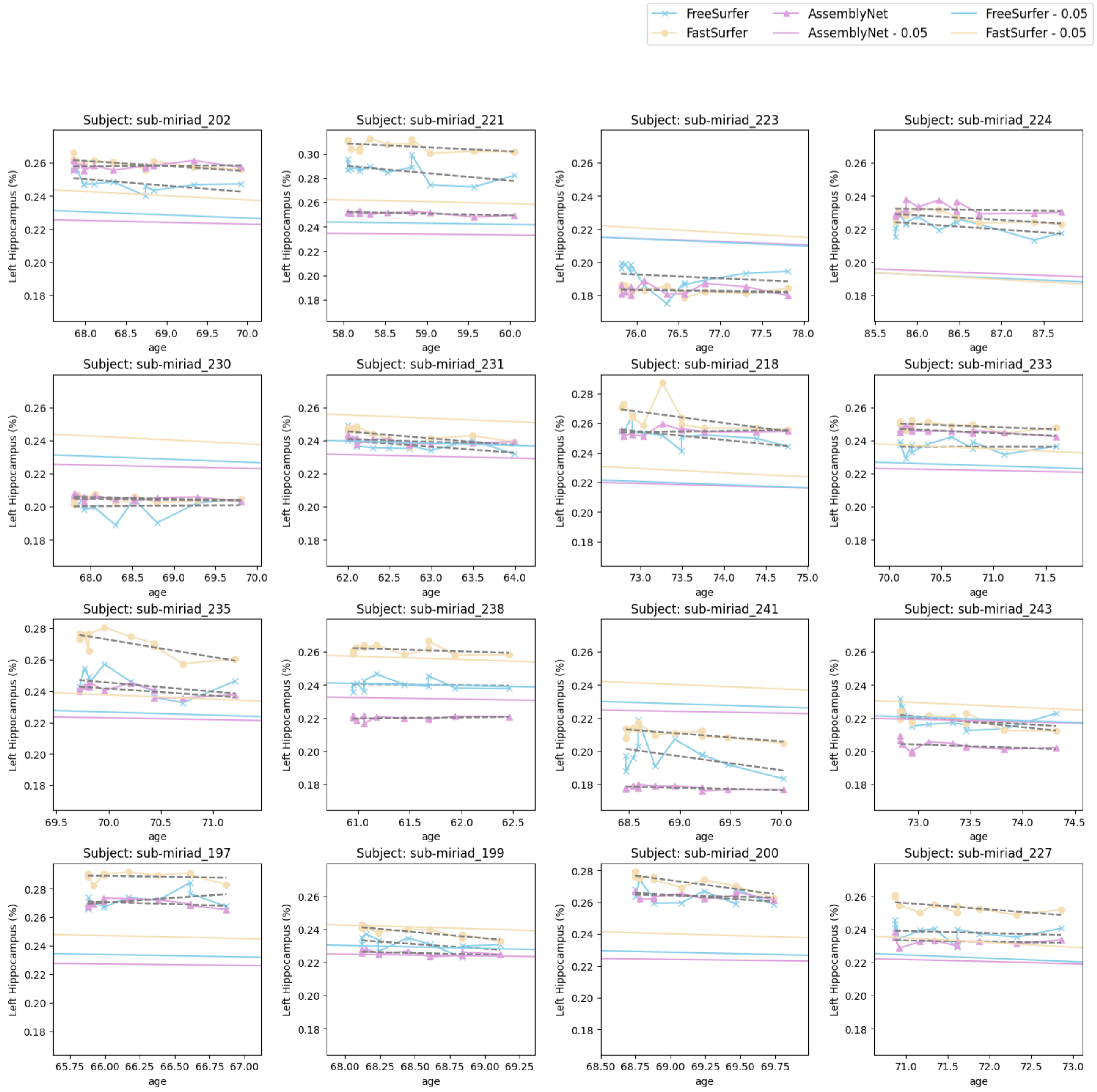

Supplementary Figure S15: Visualization of the evolution of left hippocampal volume for the 16 MIRIAD controls subjects (CN) with the most longitudinal follow-up (reference curves (5th expectile) build with 1.5T and 3T data, concave smoothing), one subject per plot

Individual AD subjects views:

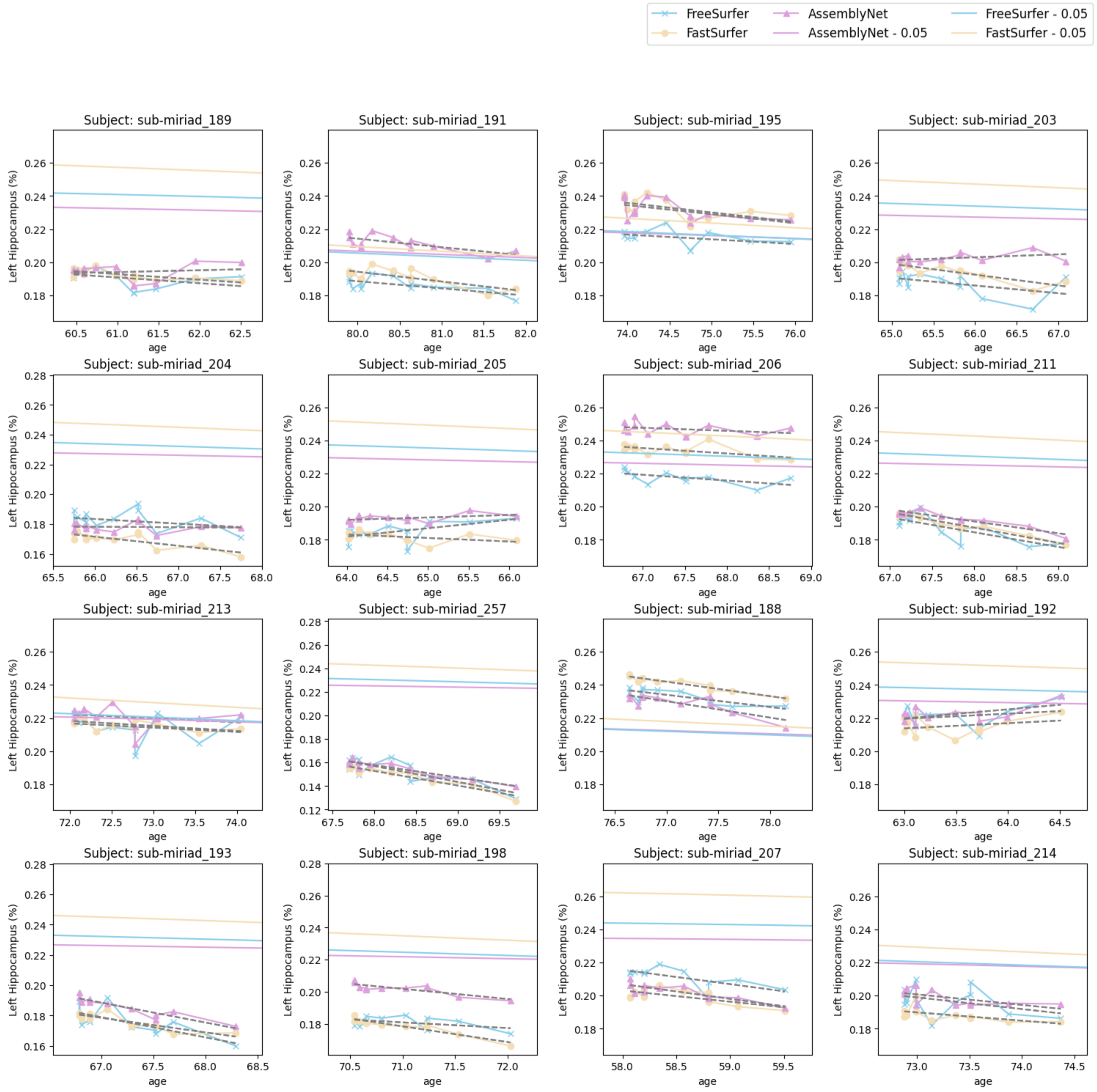

Supplementary Figure S16: Visualization of the evolution of left hippocampal volume for the 16 MIRIAD Alzheimer's patients (AD) with the most longitudinal follow-up (reference curves (5th expectile) build with 1.5T and 3T data, concave smoothing), one subject per plot

S11. ATROPHY STABILITY: INTER-SITES ANALYSIS

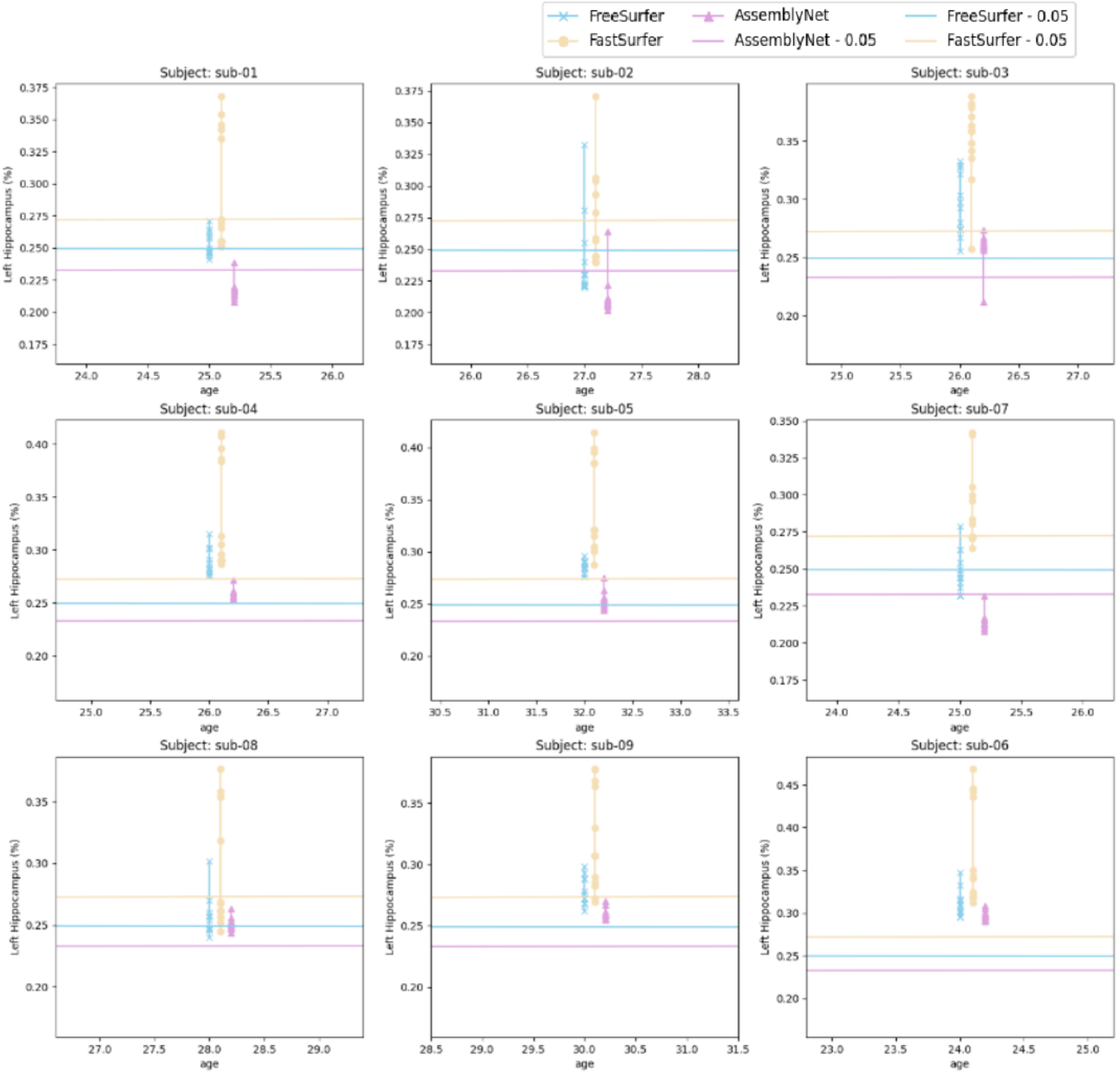

Supplementary Figure S17: Visualization of the evolution of left hippocampal volume for the 9 SRPBS traveling subjects (concave smoothing)(reference curves (5th expectile) build with 1.5T and 3T data)

Subject	AssemblyNet	FastSurfer	FreeSurfer
01	Above and below norm	Above and below norm	Above and below norm
02	Above and below norm	Above and below norm	Above and below norm
03	Above and below norm	Above and below norm	No atrophy
04	No atrophy	No atrophy	No atrophy
05	No atrophy	No atrophy	No atrophy
07	All atrophic	Above and below norm	Above and below norm
08	No atrophy	Above and below norm	Above and below norm
09	No atrophy	Above and below norm	No atrophy
10	No atrophy	No atrophy	No atrophy

Supplementary Table S2: Atrophy assessment for each subjects of SRPBS across three segmentation algorithms (reference curves build with 1.5T and 3T data), summary of Supplementary Fig. S17

S12. COMPARISON OF GAM-BASED CURVES WITH EXISTING LITERATURE

We compared our reference curves, based on GAMs, with those from the study by Coupe et al., (2017)[16]. We selected the curves from Coupe et al., (2017)[16] as they represent, to our knowledge, the only available data that allow for a direct comparison with our own curves.

In all analysis, we used the raw volumetric outputs generated by AssemblyNet. Although AssemblyNet includes options (-age and -sex) to generate individualized normative reports, these options compare the subject's raw volumetric outputs to precomputed normative reference bounds as described in Coupe et al., (2017)[16].

The reference curves from Coupe et al., (2017)[16], derived from 2,944 healthy subjects and generated using AssemblyNet with a different modeling approach, are compared here to our own GAM-based curves. The goal is to assess the impact of segmentation algorithm, statistical model, and dataset choice, focusing on hippocampal volumes. In Coupe et al., (2017)[16], reference curves were generated using region-specific models selected based on statistical relevance. These models include hybrid forms (e.g., exponential growth followed by a polynomial decline), third-order polynomials (cubic), second-order (quadratic), and linear models. For example, a cubic model was selected for the caudate and a quadratic model for the putamen and the hippocampus.

Supplementary Fig. S18 shows that our AssemblyNet-based curves closely match those from Coupe et al., (2017)[16], which is expected since both approaches use the same segmentation algorithm. In contrast, the curves generated with FastSurfer show the greatest divergence; the 5th expectile curve even exceeds the upper bound of the reference curves from Coupe et al., (2017)[16].

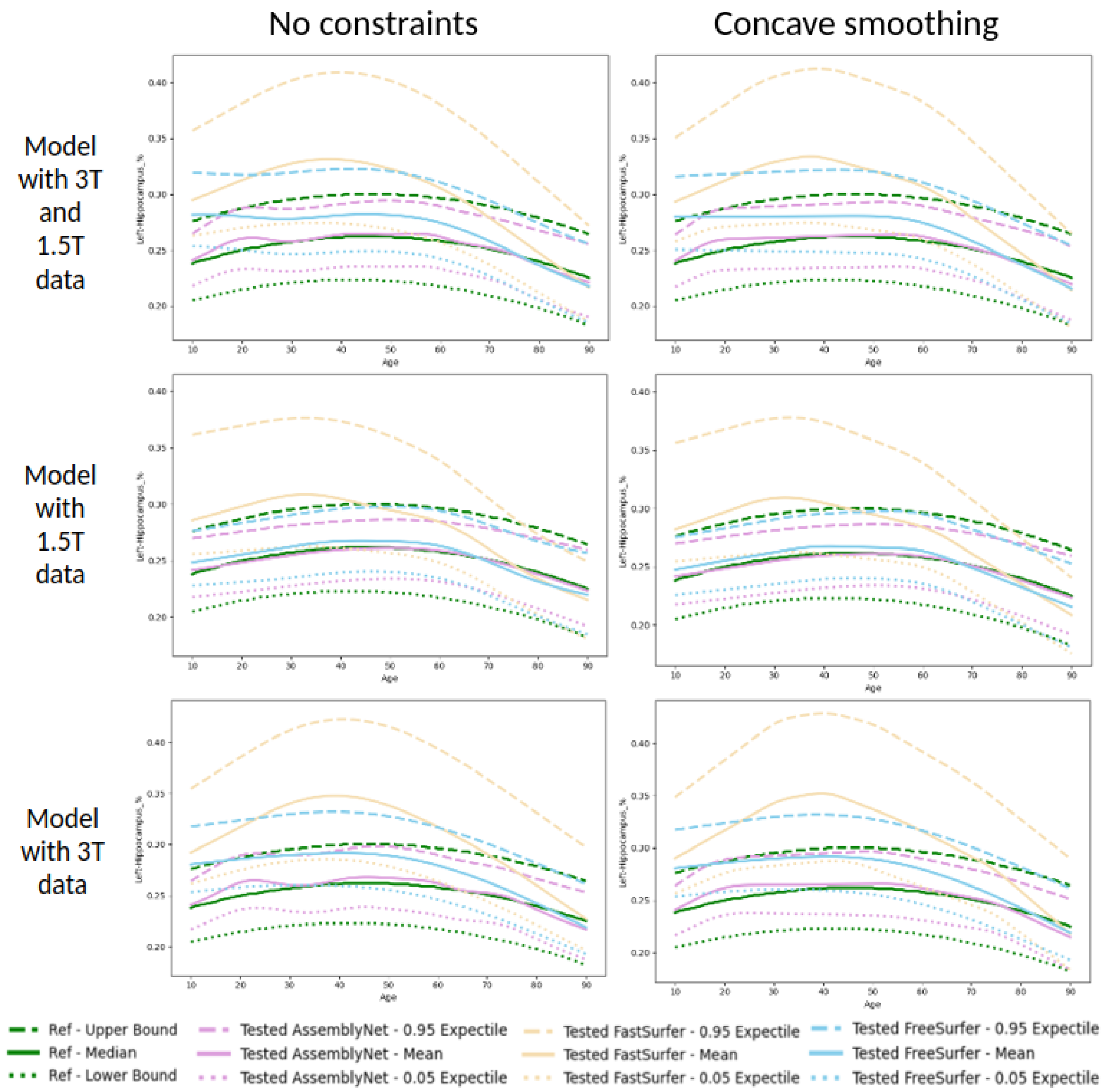

Supplementary Figure S18: The reference curves from Coupe et al., (2017)[16] (in green) for the left hippocampus are compared to those obtained using AssemblyNet (in purple), FastSurfer (in yellow), and FreeSurfer (in blue). These curves were generated using GAMs, based on the full dataset (combined 1.5T and 3T scans) with normalized volumes. The 5th (red), 50th (green), and 95th (red) expectiles are shown to characterize population variability across the lifespan. The left column shows curves without concavity constraints, while the right column presents curves with concave smoothing.

The reference curves obtained with AssemblyNet, FastSurfer, and FreeSurfer for the left hippocampus were compared to those from Coupe et al., (2017)[16] using various error metrics (sMSPE, sMAPE, wMAPE, and sMdAPE) computed for the lower, median, and upper curves (Supplementary Fig. S19).

AssemblyNet shows the best performance across all metrics (sMSPE, sMAPE, wMAPE, sMdAPE), with consistently lower errors than those observed for FastSurfer and FreeSurfer.

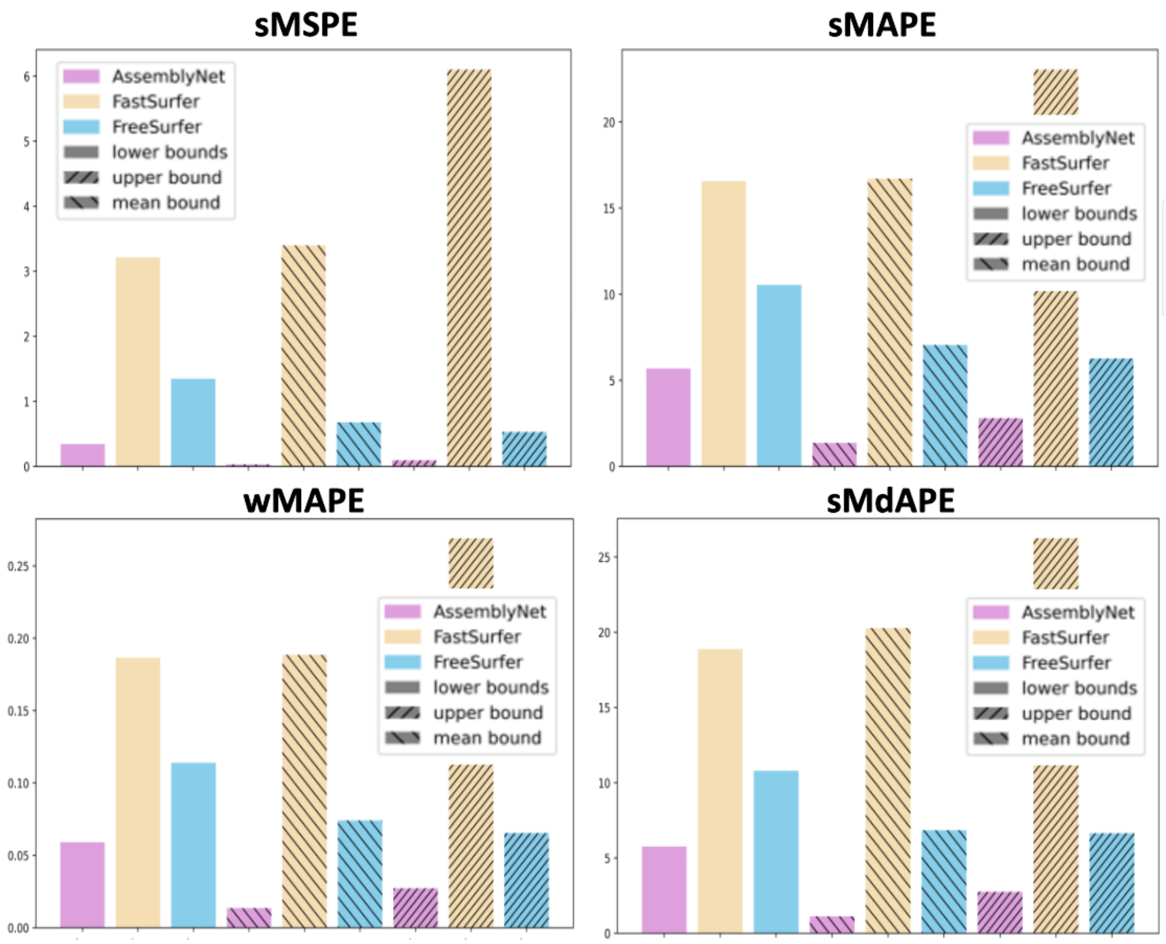

Supplementary Figure S19: Error metric values (sMSPE (symmetric Mean Squared Percentage Error), sMAPE (symmetric Mean Absolute Percentage Error), wMAPE (weighted Mean Absolute Percentage Error), and sMdAPE (symmetric Median Absolute Percentage Error)) for AssemblyNet, FastSurfer, and FreeSurfer for the left hippocampus, relative to the literature-based reference curves, computed for the lower, median, and upper curves.

**Subjects**	**AssemblyNet**	**FastSurfer**	**FreeSurfer**
AD subjects	63.01% / 59.36%	66.21% / 63.47%	68.49% / 63.93%
CN subjects	6.27% / 4.31%	2.35% / 2.35%	3.92% / 2.74%

Supplementary Table S3: Left / right hippocampal atrophy percentages (for each cell: value1: left / value2: right) computed for the Alzheimer's disease ("AD") and cognitively normal ("CN") groups using the reference curves from Coupe et al., (2017)[16], applied to the same ADNI dataset used in the previous analysis.

REFERENCES

[1] Jadon, A., Patil, A. & Jadon, S. A comprehensive survey of regression-based loss functions for time series forecasting. In *International Conference on Data Management, Analytics & Innovation*, 117–147 (Springer, 2024).

[2] Saigal, S. & Mehrotra, D. Performance comparison of time series data using predictive data mining techniques. *Advances in Information Mining* **4**, 57–66 (2012).

[3] Fan, W. D. *et al.* Bicycle volume: Counting machine validation & correction, estimating & forecasting, and analysis of injury risk. Tech. Rep., North Carolina Department of Transportation. Research and Development Unit (2021).

[4] Vanne, J., Aho, E., Hamalainen, T. D. & Kuusilinna, K. A high-performance sum of absolute difference implementation for motion estimation. *IEEE transactions on circuits and systems for video technology* **16**, 876–883 (2006).

[5] Eidous, O. M. & Ananbeh, E. Approximations for cumulative distribution function of standard normal. *Journal of Statistics and Management Systems* **25**, 541–547 (2022).

[6] Tazikeh, S. *et al.* A systematic and critical review of asphaltene adsorption from macroscopic to microscopic scale: Theoretical, experimental, statistical, intelligent, and molecular dynamics simulation approaches. *Fuel* **329**, 125379 (2022).

[7] Chai, T. & Draxler, R. R. Root mean square error (rmse) or mean absolute error (mae)?–arguments against avoiding rmse in the literature. *Geoscientific model development* **7**, 1247–1250 (2014).

[8] Willmott, C. J. & Matsuura, K. Advantages of the mean absolute error (mae) over the root mean square error (rmse) in assessing average model performance. *Climate research* **30**, 79–82 (2005).

[9] Massmann, C. & Holzmann, H. Analysing goodness of fit measures using a sensitivity based approach. In *EGU General Assembly Conference Abstracts*, 12354 (2012).

[10] Mir, A. A. *et al.* An improved imputation method for accurate prediction of imputed dataset based radon time series. *Ieee Access* **10**, 20590–20601 (2022).

[11] Hyndman, R. J. & Koehler, A. B. Another look at measures of forecast accuracy. *International journal of forecasting* **22**, 679–688 (2006).

[12] Lai, T., To, W. M., Lo, W. & Choy, Y. S. Modeling of electricity consumption in the asian gaming and tourism center—macao sar, people's republic of china. *Energy* **33**, 679–688 (2008).

[13] De Myttenaere, A., Golden, B., Le Grand, B. & Rossi, F. Mean absolute percentage error for regression models. *Neurocomputing* **192**, 38–48 (2016).

[14] Wang, X., Peng, Y. & Ma, W. An end-to-end smart predict-then-optimize framework for vehicle relocation problems in large-scale vehicle crowd sensing. *arXiv preprint arXiv:2411.18432* (2024).

[15] Kreinovich, V., Nguyen, H. T. & Ouncharoen, R. How to estimate forecasting quality: A system-motivated derivation of symmetric mean absolute percentage error (smape) and other similar characteristics. *Departmental Technical Reports (CS). 865* (2014).

[16] Coupé, P., Catheline, G., Lanuza, E., Manjón, J. V. & Initiative, A. D. N. Towards a unified analysis of brain maturation and aging across the entire lifespan: A mri analysis. *Human brain mapping* **38**, 5501–5518 (2017).
